# *Mycobacterium tuberculosis* complex genotypes circulating in Nigeria based on spoligotyping obtained from Ziehl-Neelsen stained slides extracted DNA

**DOI:** 10.1371/journal.pntd.0006242

**Published:** 2018-02-15

**Authors:** Barbara Molina-Moya, Michel K. Gomgnimbou, Lizania Spinasse, Joshua Obasanya, Olanrewaju Oladimeji, Russell Dacombe, Thomas Edwards, Xavier-Olessa Daragon, Lovett Lawson, Saddiq T. Abdurrahman, Luis E. Cuevas, Jose Dominguez, Christophe Sola

**Affiliations:** 1 Servei de Microbiologia, Hospital Universitari Germans Trias i Pujol, Fundacio Institut d'Investigació Germans Trias i Pujol. Universitat Autònoma de Barcelona. CIBER Enfermedades Respiratorias, Badalona, Spain; 2 Institut for Integrative Biology of the Cell (I2BC), CEA, CNRS, Univ. Paris Sud, Université Paris-Saclay, Gif-sur-Yvette, France; 3 Centre Muraz, and Faculté de Médecine, Bobo-Dioulasso, Burkina Faso; 4 National TB, Buruli Ulcer and Leprosy Control Programme, Abuja, Nigeria; 5 Liverpool School of Tropical Medicine, Pembroke Place, Liverpool, United Kingdom; 6 Zankli Medical Center, Abuja, Nigeria; 7 Bingham University, Nassarawa State, Nigeria; University of Liverpool, UNITED KINGDOM

## Abstract

Tuberculosis (TB) remains one of the most threatening diseases and Nigeria has one of the world’s largest burdens. This study performed high-throughput spoligotyping directly on sputum smears to describe the *Mycobacterium tuberculosis* strains circulating among new TB patients in Nigeria.

**Methods:**

All State TB control programmes in Nigeria were requested to submit 25–50 smear-positive Ziehl-Neelsen (ZN) stained slides for screening during 2013–2014. DNA was extracted from 929 slides for spoligotyping and drug-resistance analysis using microbead-based flow-cytometry suspension arrays.

**Results:**

Spoligotyping results were obtained for 549 (59.1%) of 929 samples. Lineage 4 Cameroon sublineage (L4.6.2) represented half of the patterns, *Mycobacterium africanum* (L5 and L6) represented one fifth of the patterns, and all other lineages, including other L4 sublineages, represented one third of the patterns. Sublineage L4.6.2 was mostly identified in the north of the country whereas L5 was mostly observed in the south and L6 was scattered. The spatial distribution of genotypes had genetic geographic gradients. We did not obtain results enabling the detection of drug-resistance mutations.

**Conclusion/Significance:**

We present the first national snapshot of the *M*. *tuberculosis* spoligotypes circulating in Nigeria based on ZN slides. Spoligotyping data can be obtained in a rapid and high-throughput manner with DNA extracted from ZN-stained slides, which may potentially improve our understanding of the genetic epidemiology of TB.

## Author summary

Using a classical genotyping method designated as “spoligotyping”, which targets polymorphic repetitive DNA loci, we present a general snapshot of the genetic diversity of *Mycobacterium tuberculosis* complex causing tuberculosis in Nigeria. Our results were obtained on a collection of 549 DNAs, extracted from Ziehl-Neelsen stained sputum smears gathered and representative from 36 Nigerian states and during 2013–2014. We show the ubiquitous presence on the Nigerian territory of a sublineage of Lineage 4, designated as L4.6.2 or “Cameroon” Lineage, which represents almost 50% of all patterns, more prevalent where *Mycobacterium africanum* west African 1 (Lineage 5) is absent. We also show that Lineage 5 is geographically linked to the south-east of the country, where it is the most prevalent, and represents approximately 20% of all the samples. The last third is linked to all other L4 sublineages and to L6 (*M*. *africanum* west African 2). Our results confirm the strong phylogeographical structure of *Mycobacterium tuberculosis* complex in Nigeria and are suggestive of a long-term coevolution history between *Homo sapiens sapiens* and *Mycobacterium tuberculosis* complex.

## Introduction

In 2015, six countries, Nigeria, India, Indonesia, China, Pakistan and South-Africa, accounted for 60% of all TB cases in the world, with Nigeria having the second largest TB burden in Africa, with an estimated 586,000 cases in 2015 [1]. Nigeria is also among the ten countries with the largest gaps between notifications of new and relapse (incident) TB cases and the best estimates of TB incidence in 2016 [2]. Despite the large number of cases, there is a paucity of information of the genetic diversity of *Mycobacterium tuberculosis* complex (MTC) circulating in the country and in west Africa [3–7]. In a previous study, we reported the genetic diversity of MTC in three Nigerian cities (Abuja, Ibadan and Nnewi) using a CRISPR-based fingerprinting method (spoligotyping) based on DNA extracted from clinical isolates [5, 8, 9]. Lineages 4.6.2 (Cameroon family) and Lineages 5 and 6 (*Mycobacterium africanum* west African 1 and 2) were the main lineages found [5]. The study however reported significant variations between the cities and it was not representative of whole Nigeria. Although it would be desirable to conduct genetic diversity studies on MTC cultures, with a wider geographical representation of patients, this would require considerable resources and would be logistically complex. Hence, in a large country like Nigeria, obtaining a representative set of cultures from all inhabited regions is difficult to achieve.

It has been reported that it is possible to conduct spoligotyping analysis directly from Ziehl-Neelsen (ZN) stained slides, scratching the material from the slides and extracting the DNA [10]. However the method has a relatively poor sensitivity and has not gained wide acceptance as a direct molecular identification technique [10].

Despite this limitation, direct spoligotyping from smears would be logistically simpler and facilitate studies with wider geographical representation, bypassing the need for culture, and allowing the direct identification of *M*. *africanum* (L5 and L6), which has specific metabolic requirements and is difficult to grow [11, 12]. In one recent study we demonstrated that the apparent disappearance of *M*. *africanum* from Burkina Faso was a sampling artefact [13, 14] and other teams in Cameroon and Benin are currently facing the dilemma on whether *M*. *africanum* is truly disappearing or not, and a study in Ghana has shown that *M*. *africanum* still represents 20% of all TB cases [15]. Indeed, an alternative explanation would be the progressive replacement of *M*. *africanum* by more modern (Lineage 4) isolates [14].

The aim of this study was to conduct a nation-wide description of the diversity of tuberculosis genotypes based on sputum smear extracts, to assess the global geographical genetic structure of MTC in Nigeria. In addition, we tested a new Nucleic Acid Amplification test (NAAT) method «TB-SPRINT», that allows simultaneous spoligotyping and prediction of drug-resistance in a sample collection based on ZN extracted DNA [16–18].

## Materials and methods

### Sample collection and DNA extraction

Since we wanted to provide a detailed spatial analysis of circulating MTC genotypes, we studied *M*. *tuberculosis* diversity in 36 states using ZN-stained smears representative of the country to build geographical genetic maps. A total of 929 ZN slides with grades 3+ (n = 505) and 2+ (n = 424), were collected from adults attending health facilities with cough of more than two weeks duration who had not received treatment for TB [19]. Samples were gathered as part of routine clinical work for the diagnosis of TB. Slides processed by the laboratories were then selected for the routine external quality assurance (EQA) activities conducted by the National TB and Leprosy Control Program (NTLCP) to evaluate the quality of smear microscopy in each State. These slides are routinely anonymised at the time they are submitted to the reference laboratory. Smear positive samples received by the reference laboratory for blind rechecking as part of the EQA were then selected for the study. Only date and origin of the sample were collected. Subjects did not provide informed consent as this is a routine diagnostic procedure. The Research Ethics Committee of the Liverpool School of Tropical Medicine and the Institutional Review Board of Zankli Medical Centre approved the study protocol.

ZN slides were requested via the State TB focal person of the NTLCP from all 36 States and the Federal Capital Territory of Nigeria during the years 2013 and 2014. Slides were shipped by post to Zankli Medical Centre in Abuja. ZN staining and smear grading were performed by the routine diagnostic laboratories following national guidelines [20]. DNA extraction was performed from the stained slides as follows: the mineral oil was removed with xylene, 25 μl of filtered TRIS-EDTA was added to the slides and the material was scraped off into a microcentrifuge tube. The tubes were shipped at room temperature to the Hospital Universitari Germans Trias i Pujol (Spain), where 75 μl of Chelex suspension was added. After thorough mixing, samples were incubated for 30 minutes at 95°C, sonicated for 5 minutes and centrifuged for 15 minutes at 14,000 g at 4°C. The supernatant was transferred to fresh microcentrifuge tubes and sent to the Institute for Integrative Cell Biology (Gif-sur-Yvette, France) for high-throughput spoligotyping.

### Spoligotyping

High-throughput spoligotyping was performed using the microbead-based method using flow-cytometry suspension arrays (Luminex 200, Luminex Corp, Austin, TX), as previously described [21]. TB-SPOL and TB-SPRINT kits were purchased from Beamedex (Beamedex, Orsay, France; www.beamedex.com). Given the low DNA content of the samples, the PCR protocol was adjusted by increasing PCR cycles from 20 to up to 35 cycles. Each spoligotyping profile was verified at least twice and doubtful profiles were rejected. Quality control was conducted by three experts (MKG, BMM, CS) who independently assessed individual spoligotyping profiles. Full or empty squares represents the presence or absence of the 43 classical CRISPR spacers. The spoligotyping patterns were further labelled using the SITVITWEB database [22]. Lineages (L1 to L7) and sub-lineages (such as L.4.6.2, the Cameroon Lineage) were designated using the latest taxonomical standards as provided by whole-genome sequencing, using published lineage definition [23, 24]. When available, the lineage designation was complemented by the classical spoligotyping-based clade designation, e.g. Lineage 4.6.2 is also designated as the «Cameroon Clade (CAM)» [25].

### Clustering analysis and Bioinformatical analysis

**Fig 1:**
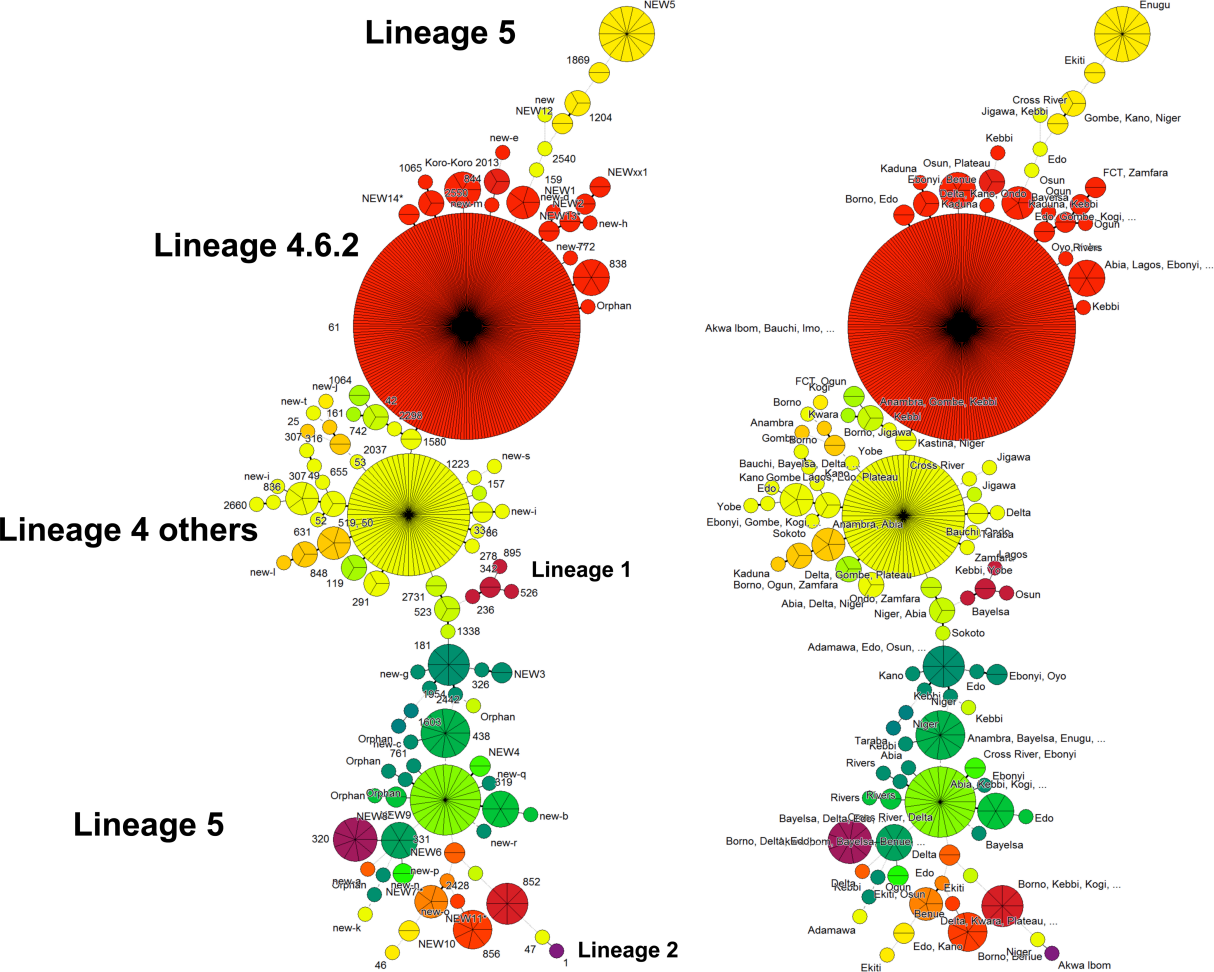
Minimum Spanning tree built on 549 spoligotypes found in Nigeria. Main Lineages are labelled with font size relative to their prevalence. On the right side of the Figure, precise geographic assignation of genotypes (e.g. NEW 5, n = 13 in Enugu state).

An Excel result file was imported into Bionumerics version 7.5 (Biomérieux, Applied Maths, St Martens-Latem, Belgium). A minimum spanning tree (MST) was built using the Bionumerics user’s manual (Fig 1).

### Map building and spatial representation

A Mac OSX Version of QGIS (v2.18 Las Palmas de Gran Canaria) was downloaded and installed from: http://www.kyngchaos.com/software/qgis. A licence-free Nigerian map with administrative level 1 was downloaded as a shapefile from http://maplibrary.org/library/stacks/Africa/Nigeria/index.htm; Latitude and longitude of the main Nigerian cities was downloaded from http://www.downloadexcelfiles.com/wo_en/download-list-latitudelongitude-cities-nigeria#.WUPbwYXSWDQ and http://simplemaps.com/data/world-cities.

Nigerian Population records and density was obtained by collecting data from: http://www.iplussolutions.org/isolutions-leads-consortium-streamline-patient-access-essential-treatments-nigeria-0 (assessed on November 2017, 20th) and https://www.citypopulation.de (assessed on December 2017, 8th).

**Fig 2:**
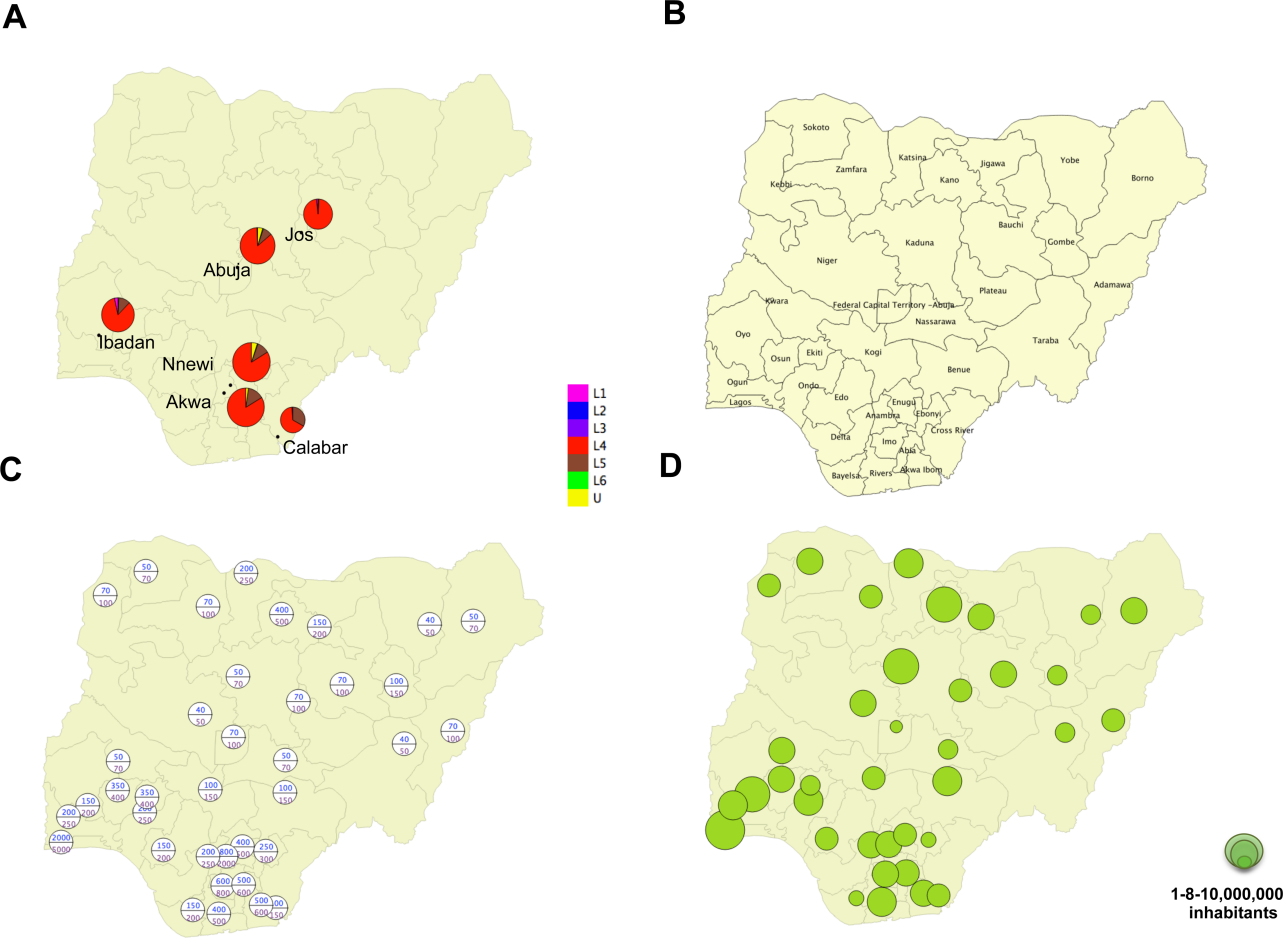
(A) Spatial distribution shown as Apple-pie charts of L1-L6 *M*. *tuberculosis* complex (MTC) genotype prevalence from previous published studies performed in Nigeria; Plateau state:[3]; Abuja:[5]; Anambra state:[5,26]; Oyo state: [5]; Cross-River state: [4]; (B) Administrative map of Nigeria (Level 1 administrative division since 1996); (C) Population density (min-max) by state (cf. S2 Table for raw data); (D) population size by State (cf. S2 Table for raw data).

**Fig 3:**
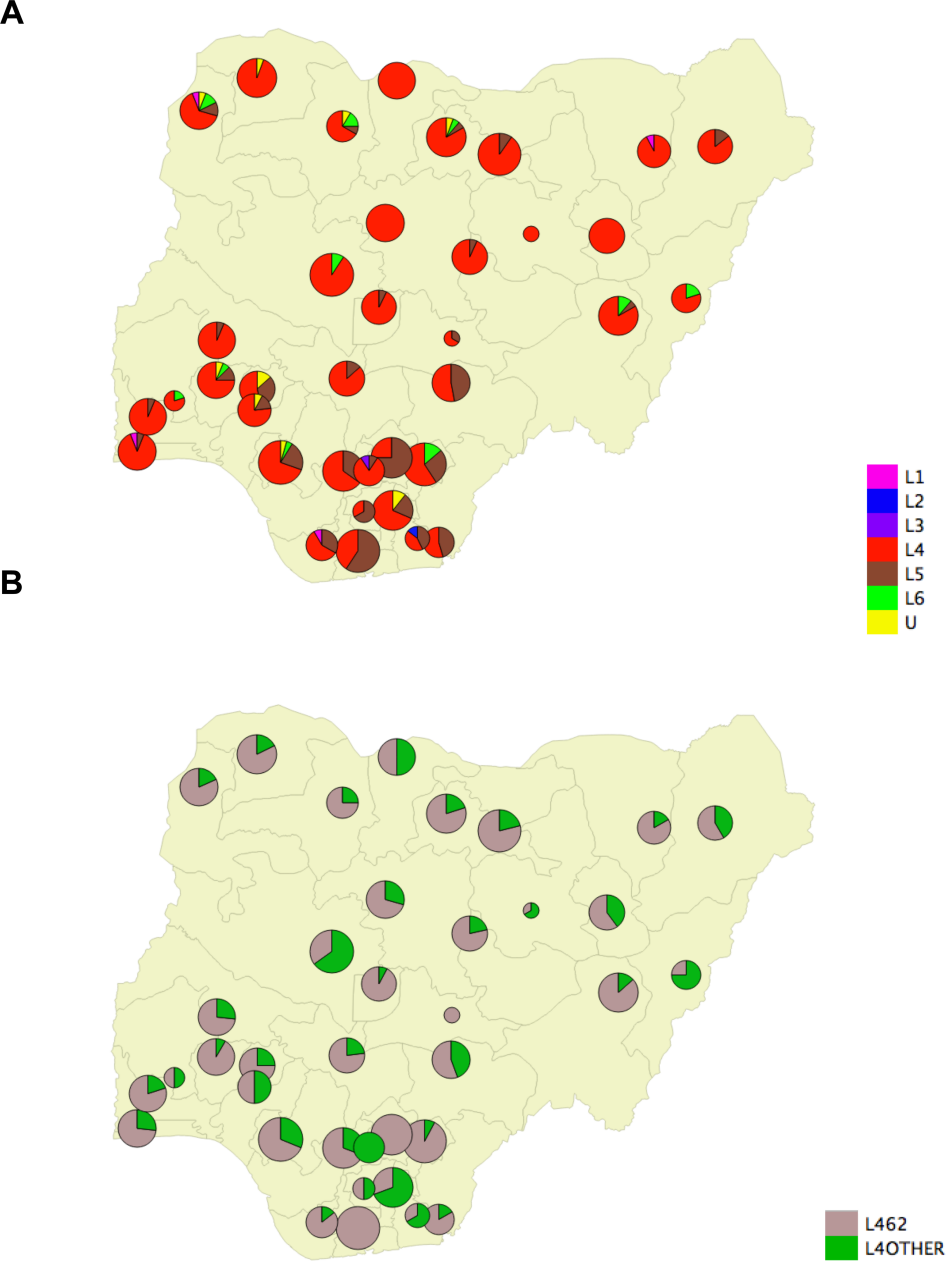
(A) Spatial Distribution shown as Apple-pie charts of L1-L6 *M*. *tuberculosis* complex (MTC) genotype prevalence on 549 positive ZN-extracted DNA; (B): Spatial Distribution shown as Apple-pie charts of MTC Lineage 4.6.2 (Cameroon sublineage) within the L4 sublineage. (cf S2 Table for raw data).

Maps (Fig 2 and Fig 3) were produced using QGIS after importation of.shp and.csv files using the user’s manual (cf. S2 Table).

## Results

### Global genetic diversity of *M*. *tuberculosis* complex in Nigeria

*M*. *tuberculosis* spoligotyping results were obtained in 549 of 929 smear samples (genotyping rate = 59.1%). Of these, 327 (64.8%) were obtained of the 505 slides with smear grades 3+ and 222 (52.4%) of the 424 slides with smear grades 2+. Spoligotyping patterns were named using Spoligotyping International Type (SIT) tags (S1 Table). An MST tree that describes the global population structure is shown in Fig 1.

One hundred and two different spoligotyping patterns were observed, among which 55 had unique patterns with one sample and 47 were clusters with two or more samples. The global distribution of patterns is dominated by the «Cameroon» family, Lineage 4.6.2, including SIT61 and derived signatures characterized by the absence of spacers 23–25 and 33–36, representing about 50% of patterns, and *M*. *africanum*, which included *M*. *africanum* west African 1 (L5; SIT431 and SIT338, signature: absence of spacer 8–12 and 37–39) and *M*. *africanum* west-African 2 (L6; SIT181, signature: absence of 8–9 and 39), which represented approximately 20% of patterns. All other lineages L1 to L4, including East African India/EAI (L1, absence of spacers 29–32 and 34), Beijing (L2, absence of spacers 1–34), Central Asia/CAS (L3, absence of spacers 4–7 and 23–34), Euro-American (L4, absence of spacer 33–36), i.e. all «T spoligotypes» and other derived sublineages (Haarlem, LAM, S, and others, absence of spacers 33–36 plus specific signatures), represented circa 30% of all patterns.

### Spoligotyping-based cluster analysis

The main spoligotyping results are shown in S1 Table with Lineage assignation. Fig 1 shows the cluster analysis. Two hundred and eighty six (52.1%) of the 549 samples belonged to the L4.6.2 (Cameroon Family), of which 278 were found in 13 different clusters (SIT61, n = 232; SIT838, n = 6; SIT844, n = 3; SIT852, n = 8; SIT1204, n = 3; SIT2550, n = 3; NEW “Koro-Koro 2013”, n = 6 (described in Cameroon as CAM57); «NEW1», n = 4; «NEW12», n = 2; «NEW13*», n = 5; «NEW14*», n = 2; «NEW2», n = 2; «new-f*», n = 2), and eight patterns were orphan with a classical L4.6.2. signature. Full results are shown in S1 Table and S2 Table.

The L5 (*M*. *africanum* west African 1) genetic diversity included 87 (15.8%) isolates, of which 75 were identified in 11 clusters (SIT319, n = 6; SIT320, n = 9; SIT331, n = 23; SIT438, n = 11; SIT856, n = 7; «NEW11*», n = 5; «NEW4», n = 2; «NEW6», n = 2; «NEW7’*», n = 2; «NEW8*», n = 6; « NEW9 », n = 2), and 12 were orphan. The L6 (*M*. *africanum* west-African 2) included 15 (2.7%) samples of which 10 were found in two clusters (SIT181, n = 8; «NEW3», n = 2) and five were orphan.

The L4 (T1 subfamilies, i.e. other than L4.6.2 and L4.6) included 82 samples among which 75 were found in four clusters (SIT53, n = 68; SIT291, n = 3; SIT334, n = 2; SIT1580, n = 2) and seven were orphan. Within the T2 subfamily (Lineage 4.6), 12 samples were identified in three clusters (SIT52, n = 5; SIT742, n = 2; SIT848, n = 3) and two patterns were orphan. Lastly, a single pattern of the T3 subfamily and four additional orphan patterns were classified as belonging to the broad L4 lineage. Thirteen samples were classified as H3 (L4.1.2 or L4.5) [24]. Nine of these samples were found in three clusters (SIT49, n = 5; SIT50, n = 2; SIT307, n = 2) and four patterns were orphan. A total of seven samples were classified as belonging to Lineage 4.3 (LAM), of which five were in two LAM9 clusters (SIT42, n = 3; SIT1064, n = 2), one was orphan, and one was classified as LAM3.

Twenty samples were classified as Unknown («U»), among which 16 were found in three clusters (SIT1869, n = 2; «NEW10», n = 2; «NEW5», n = 14) and two were orphan patterns.

Finally, the genetic diversity of the remaining 19 samples was as follows: L1 (EAI5; n = 2), L1 (EAI2_Manilla; n = 1); L2 (Beijing; n = 1), L3 (CAS1_Delhi; n = 1), L4.1.1.2 (X, SIT119; n = 3), Lineage 4 (Manu_ancestor SIT523; n = 3) L6 (*Mycobacterium bovis*; n = 2), and five orphan patterns with no SIT or family that could be assigned by spoligotyping.

### Clustering analysis by Minimum Spanning Tree

Fig 1 presents a Minimum Spanning Tree of all spoligotyping results. Relative diameters of clusters represent the relative percentage of each spoligotyping cluster. Clonal complexes, i.e. samples historically linked to a progenitor clone are easily identified and we observed that L.4.6.2 predominates. The other main clusters presented, belonging to L4 and L5 are also mentioned. The relative position of some clusters of L5 on top of Fig 1 is misleading since these clusters are not related to L4. The States information for the clustered isolates (Fig 1, right panel) shows both intra-State and inter-State clusters.

### Geographical distribution of *M*. *tuberculosis* complex lineages in Nigeria

Fig 2A presents previous results in settings where investigations had been done. Fig 2B shows the list of States of Nigeria. Fig 2C and 2D described population density and absolute population data respectively (see also S2 Table). Fig 3A shows the relative percentage of L1-L6 by State. L4.6.2 was found in 36 out of 37 States (all except Anambra State). L5 was found in 28 States (23 in the south and 5 in the north of the country). L6 was found in 10 States throughout the country but underrepresented in the south, where L5 predominates. Fig 3A and 3B show that there is a predominance of L4.6.2 in the north and a predominance of L5 in the south of the country. In six states, all in the north, central, or west (Yobe, Gombe, Bauchi, Katsina, Kaduna, Niger and Sokoto) we did not detect any *M*. *africanum*. The spatial distribution of L4.6.2 in Fig 3B shows that this lineage is prevalent everywhere in the country. However, within L4, L4.6.2 was relatively less frequent compared to other L4 sublineages where L5 predominates.

L5 was present in 28 out of 37 states and was most prevalent in the south and south-east, with Enugu (75%, 15/20) Imo (66%, 4/6) Rivers (60%, 13/22), Benue (47%, 8/17), Akwa Ibom (42%, 3/7), Delta (35%, 7/20), Ebonyi (27%, 6/22) and Abia States (21%, 4/19), in decreasing order, and a marked south-east tropism (Fig 3A), with Ekiti (33%, 5/15) and Ondo (15%, 2/13) being the two most south-western states where L5 clusters were detected. Rivers was the only state with three distinct L5 clusters (SIT430, SIT331 and NEW11). Benue and Enugu States show the presence of relatively large L5 clusters (SIT856 (n = 6) and NEW5 (n = 13), respectively). L6 was found scattered from north to south and east to west in 10 states, detected at low prevalence and only in the Ebonyi state in the south-east. In three states in the north (Kebbi, Zamfara, Kano) and four in the south (Osun, Edo, Abonyi, Taraba) both L5 and L6 cases were detected.

## Discussion

Nigeria is one of the most ethnically diverse and demographically and economically active countries in Africa with an estimated population of 173 million in 2013 and a life expectancy of 55 years (2013) [26]. It is also a Federal country with a rich historical, cultural, ethnical, economical and anthropological diversity and many local languages. Nigerian *M*. *tuberculosis* genetic diversity had never been investigated at a national scale and recent publications attempting to describe the molecular epidemiology of TB included only a selection of States (e.g. Anambra, Cross River, Oyo, Plateau, and the FCT) [4, 5, 27–29]. Our study confirms that L4.6.2 is spread over the whole country. Further whole genome sequencing (WGS) would also allow to estimate divergence times and phylogeny within and between sublineages by estimating molecular clock rates based on single nucleotide polymorphisms (SNPs) numbers detected [30, 31].

These new results confirm our previous quantitative results obtained on MTC Lineages prevalence in Anambra, FCT and Oyo states and extends it to the whole Nigerian territory [32]. It is also evident that the L4.6.2 sublineage is not limited to Cameroon and has a much wider geographic distribution [25, 33]. Our results suggest that L6.4.2 is spreading and hence should now be the focus of WGS studies in Nigeria, whether for molecular epidemiology or to gain further knowledge on the resistome and evolutionary history of this L4 sublineage.

We found a relatively high percentage of L5-L6, especially in the south-east of the country, compared to neighbouring countries such as Cameroon and Benin, where it has been suggested that L5-L6 were disappearing [12, 34]. L5-L6 are geographically restricted and clustered in some states, especially in the south-east, where it represents an important percentage of cases. When comparing to neighbouring Benin and Cameroon on the west and east Nigerian borders respectively, the TB genetic structure is quite different. In Benin, a recent study of 100 isolates recruited in 2014, reported the following distribution: L1: 0%, L2: 8%, L3: 1%, L4: 77% (46% of L.4.6.2-CAM and 31% of others), L5: 12%, L6: 2% [34]. If we take into account the most recent results obtained, there might be an underestimation of L5 in the Benin study due to difficulty to grow *M*. *africanum* [35]. In Cameroon, the prevalence of *M*. *africanum* would have dropped in 2004–2005 to 3.3% [12]. Another more recent study performed in 2009 on 509 patients in the Adamaoua region reports 2,3% of *M*. *africanum* [36].

L5 clones (SIT438, SIT331, SIT319) are known to be more prevalent in countries around the Gulf of Guinea [6, 34, 37]. *M*. *africanum* prevalence remains stable in Ghana and it has been found significantly more common in patients of the Ewe ethnic group in this country [38]. Furthermore, there seems to be a high diversity of *M*. *africanum* in Ghana [39], which was not the result of a single outbreak, as the spoligotyping patterns from Ewe patients were quite diverse [38]. In this paper, we do not provide a formal analysis such as spatial regression proving the link of L5 to specific human subpopulations. Nevertheless, we suggest linguistic, ethnical and cultural association of some MTC genotypes (Lineage 5) with specific populations in Nigeria, that shows, as it had been shown in Ghana, a long-lasting co-evolution of *M*. *tuberculosis* and *Homo sapiens sapiens* [15, 40].

Recently, a study described 315 TB cases caused by *M*. *africanum* (L5-L6) in the USA during 2004–2013 [41]. Half of the L5 cases were linked to Nigerian-born patients [41]. We first tagged these spoligotypes using SITVITWEB [22]. Out of 305 patterns, 172 got a SIT label. Common clusters as well as seven new clusters were found to be shared between the USA 2004–2013 study and this present study (S3 Table). Quite often, cases depicted as being unique (orphan) in the USA matched with clustered cases found in this study (S3 Table). These results provide clues for further epidemiological and transmission studies in relation to immigration and TB history in the USA. They also demonstrate the quality of spoligotypes obtained on sputum extracts.

The limitations of this study include that culture is more sensitive than smear microscopy. However, this is not feasible presently in most of Nigeria. Transporting sputa for cultures reduces this sensitivity due to death of mycobacteria and overgrowth of contaminating flora. We also included a low number of samples collected per State (median of 25, range 13–32), which together with the suboptimal performance of spoligotyping from ZN slides and the low number of samples with results per State (median 16, range 3–23) reduced the power of the study and the possibility to conduct a more detailed geographical analysis at sub-State level. Concerning the low sensitivity of the spoligotyping on ZN extracted material, 60–65%, the classical hot ZN is said to result in lower DNA quality than the modified cold Kinyoun method. However we did not compare the quality and quantity of DNA recovered, as the former method (hot ZN) is used throughout the study. Another limitation was the lack of results on drug-resistance-linked SNPs (*rpoB; katG*, *inhA*) after PCR amplification by TB-SPRINT on the same material, a limitation that was again likely due to a suboptimal DNA extraction method. Partial results only were obtained with poor sensitivity and paucibacillary DNA-containing material, is likely to require improved DNA extraction methods such as the selective target enrichment using specific oligonucleotide coupled microspheres or other more sophisticated DNA extraction procedures [42–43].

Despite these limitations, this study generated the first nation-wide genetic diversity study of MTC in Nigeria. In three states, Oyo, FCT, Anambra this study confirms our previous quantitative results on the L4/L5-L6 relative prevalence [5]. Our results on L5-L6 prevalence are informative of the phylogeography of *M*. *africanum* in Nigeria. In addition, our high-throughput spoligotyping technique has the potential to generate more country-wide data of the genetic distribution of MTC in countries with limited resources. This approach improves the detection of *M*. *africanum*, which is often under-represented in culture-based studies [35]. With the advent of Next Generation Sequencing (NGS), more precise population-based information could easily be obtained at affordable costs, without TB culture, even in Africa. Such projects may however require samples either with a higher DNA yield or more complex DNA extraction procedures, and necessarily result in a more selected sample of patients attending secondary and tertiary hospitals. The use of slides in turn generates genetic epidemiological information at a larger scale without the need of this infrastructure. Last but not least, our results suggest that some specific TB control and/or preventive measures could be specifically taken in densely populated areas in the south-east region of the country (Fig 2C and 2D), and that assessing the drug-resistance status of the L4.6.2 lineage is an important issue for the Nigerian TB control program.

## Supporting information

S1 Table Detailed results with key identification, laboratory numbers, experimental data: Binary and octal spoligotype, SIT (spoligotyping-international-type), positivity grade of Ziehl-Neelsen starting material, state of origin and geographic coordinates of sampling (State capital by default). (PDF)

S2 Table Country, State designations, geographic position, latitude and longitude, capital name, ACN = sample number, L1 to U (unknown): Absolute spoligotyping distribution by lineage and/or sublineage, absolute population number (by State), population density (by State), source of information. (PDF)

S3 Table Spoligotyping clusters, not found in SITVITWEB, but found between this study and Sharma *et al*. 2016 [40], with total number of isolates concerned and cluster designation. (PDF)
