# Supplementary material for: Mycobacterium tuberculosis complex genotypes circulating in Nigeria based on spoligotyping obtained from Ziehl-Neelsen stained slides extracted DNA
Source: PLoS Negl Trop Dis. 2018 Feb 15;12(2):e0006242. doi: 10.1371/journal.pntd.0006242 (PMC5831734; doi:10.1371/journal.pntd.0006242)
Supplement: S1 Table — (PDF) [file pntd.0006242.s001.pdf]

Supplementary Table 1 : Full Spoligotyping Results with key ID of samples, internal use (Rank/rack) and results : Binary, SIT tag, Octal, clade, Positivity Rate of ZN smear, Sample State origin, Latitude and Longitude

Key	Rank Number	Rack	Binary	SIT	Octal	Clade	Positivity-rate	State	Latitude	Longitude
NGA01000001-424-3	643	8	■■■	523	777777777777771	Manu_ancestor	3+	Abia	5.100397968	7.34998002
NGA01000001-482-1	644	8	■■■	523	777777777777771	Manu-ancestor	3+	Abia	5.100397968	7.34998002
NGA010000026-1561-2	657	8	■■■	523	777777777777771	Manu_ancestor	3+	Niger	9.080413431	6.01001013
NGA010000033-023-3	805	10	■■■■■■■■■■■■■■■■■■■■■■■■■■■■■■■■■■■■■■□■■■■	1338	777777777777671	Orphan	2+	Sokoto	13.06001548	5.240031289
NGA010000028-003	658	8	■■■■■■■■■■■■■■■■■■■■■■■■■■■■■■■■□■■■■■■■■■■	2731	777777777767771	Orphan	2+	Ondo	7.530430521	5.759999551
NGA010000036-710-1	858	10	■■■■■■■■■■■■■■■■■■■■■■■■■■■■■■■■□■■■■■■■■■■	2731	777777777767771	-	2+	Zamfara	12.1704057	6.659996296
NGA01000001-390-1	619	8	■■■■■■■■■■■■■■■■■■■■■■■■■■■■■■■■□□□□■■■■■■■	53	777777777760771	T1	3+	Abia	5.100397968	7.34998002
NGA01000001-477-3	620	8	■■■■■■■■■■■■■■■■■■■■■■■■■■■■■■■■□□□□■■■■■■■	53	777777777760771	T1	2+	Abia	5.100397968	7.34998002
NGA01000001-404-1	627	8	■■■■■■■■■■■■■■■■■■■■■■■■■■■■■■■■□□□□■■■■■■■	53	777777777760771	T1	3+	Abia	5.100397968	7.34998002
NGA01000001-415-1	635	8	■■■■■■■■■■■■■■■■■■■■■■■■■■■■■■■■□□□□■■■■■■■	53	777777777760771	T1	3+	Abia	5.100397968	7.34998002
NGA01000001-437-3	651	8	■■■■■■■■■■■■■■■■■■■■■■■■■■■■■■■■□□□□■■■■■■■	53	777777777760771	T1	3+	Abia	5.100397968	7.34998002
NGA01000001-495-3	652	8	■■■■■■■■■■■■■■■■■■■■■■■■■■■■■■■■□□□□■■■■■■■	53	777777777760771	T1	3+	Abia	5.100397968	7.34998002
NGA01000002-641-2	7	1	■■■■■■■■■■■■■■■■■■■■■■■■■■■■■■■■□□□□■■■■■■■	53	777777777760771	T1	3+	Adamawa	10.2703408	13.2700321
NGA01000002-267-2	14	1	■■■■■■■■■■■■■■■■■■■■■■■■■■■■■■■■□□□□■■■■■■■	53	777777777760771	T1	2+	Adamawa	10.2703408	13.2700321
NGA01000002-202-2	15	1	■■■■■■■■■■■■■■■■■■■■■■■■■■■■■■■■□□□□■■■■■■■	53	777777777760771	T1	2+	Adamawa	10.2703408	13.2700321
NGA01000002-257-2	22	1	■■■■■■■■■■■■■■■■■■■■■■■■■■■■■■■■□□□□■■■■■■■	53	777777777760771	T1	2+	Adamawa	10.2703408	13.2700321
NGA01000002-042-2	23	1	■■■■■■■■■■■■■■■■■■■■■■■■■■■■■■■■□□□□■■■■■■■	53	777777777760771	T1	3+	Adamawa	10.2703408	13.2700321
NGA01000002-047-3	31	1	■■■■■■■■■■■■■■■■■■■■■■■■■■■■■■■■□□□□■■■■■■■	53	777777777760771	T1	2+	Adamawa	10.2703408	13.2700321
NGA01000004-654-3	5	1	■■■■■■■■■■■■■■■■■■■■■■■■■■■■■■■■□□□□■■■■■■■	53	777777777760771	T1	3+	Akwa Ibom	5.007996056	7.849998524
NGA01000004-171-3	19	1	■■■■■■■■■■■■■■■■■■■■■■■■■■■■■■■■□□□□■■■■■■■	53	777777777760771	T1	2+	Akwa Ibom	5.007996056	7.849998524
NGA01000003-578-2	1	1	■■■■■■■■■■■■■■■■■■■■■■■■■■■■■■■■□□□□■■■■■■■	53	777777777760771	T1	3+	Anambra	6.210433572	7.06999711
NGA01000003-1360-2	18	1	■■■■■■■■■■■■■■■■■■■■■■■■■■■■■■■■□□□□■■■■■■■	53	777777777760771	T1	3+	Anambra	6.210433572	7.06999711
NGA01000003-687-1	33	1	■■■■■■■■■■■■■■■■■■■■■■■■■■■■■■■■□□□□■■■■■■■	53	777777777760771	T1	3+	Anambra	6.210433572	7.06999711
NGA01000003-253-2	34	1	■■■■■■■■■■■■■■■■■■■■■■■■■■■■■■■■□□□□■■■■■■■	53	777777777760771	T1	2+	Anambra	6.210433572	7.06999711
NGA01000003-1436-2	42	1	■■■■■■■■■■■■■■■■■■■■■■■■■■■■■■■■□□□□■■■■■■■	53	777777777760771	T1	2+	Anambra	6.210433572	7.06999711
NGA01000003-727-1	57	1	■■■■■■■■■■■■■■■■■■■■■■■■■■■■■■■■□□□□■■■■■■■	53	777777777760771	T1	2+	Anambra	6.210433572	7.06999711
NGA01000003-302-1	72	1	■■■■■■■■■■■■■■■■■■■■■■■■■■■■■■■■□□□□■■■■■■■	53	777777777760771	T1	2+	Anambra	6.210433572	7.06999711
NGA01000005-613-1	115	2	■■■■■■■■■■■■■■■■■■■■■■■■■■■■■■■■□□□□■■■■■■■	53	777777777760771	T1	3+	Bauchi	10.3103642	9.84000891
NGA01000006-638-2	124	2	■■■■■■■■■■■■■■■■■■■■■■■■■■■■■■■■□□□□■■■■■■■	53	777777777760771	T1	3+	Bayelsa	4.664030	6.036987
NGA01000007-750-1	94	2	■■■■■■■■■■■■■■■■■■■■■■■■■■■■■■■■□□□□■■■■■■■	53	777777777760771	T1	3+	Benue	7.729979064	8.530011351
NGA01000007-385-2	125	2	■■■■■■■■■■■■■■■■■■■■■■■■■■■■■■■■□□□□■■■■■■■	53	777777777760771	T1	3+	Benue	7.729979064	8.530011351
NGA01000006-649-3	132	2	■■■■■■■■■■■■■■■■■■■■■■■■■■■■■■■■□□□□■■■■■■■	53	777777777760771	T1	3+	Benue	7.729979064	8.530011351
NGA01000007-512-2	133	2	■■■■■■■■■■■■■■■■■■■■■■■■■■■■■■■■□□□□■■■■■■■	53	777777777760771	T1	2+	Benue	7.729979064	8.530011351
NGA01000008-023-2	621	8	■■■■■■■■■■■■■■■■■■■■■■■■■■■■■■■■□□□□■■■■■■■	53	777777777760771	T1	2+	Borno	11.84996014	13.16001298
NGA01000008-024-2	629	8	■■■■■■■■■■■■■■■■■■■■■■■■■■■■■■■■□□□□■■■■■■■	53	777777777760771	T1	2+	Borno	11.84996014	13.16001298
NGA01000010-1345-1	217	3	■■■■■■■■■■■■■■■■■■■■■■■■■■■■■■■■□□□□■■■■■■■	53	777777777760771	T1	2+	Delta	5.519958922	5.759999551
NGA01000015-1277-3	280	4	■■■■■■■■■■■■■■■■■■■■■■■■■■■■■■■■□□□□■■■■■■■	53	777777777760771	T1	3+	Gombe	10.29044293	11.16995357
NGA01000016-1025-2	283	4	■■■■■■■■■■■■■■■■■■■■■■■■■■■■■■■■□□□□■■■■■■■	53	777777777760771	T1	2+	Imo	5.492997053	7.026003588
NGA01000017-422-3	348	4	■■■■■■■■■■■■■■■■■■■■■■■■■■■■■■■■□□□□■■■■■■■	53	777777777760771	T1	3+	Jigawa	11.7991891	9.350334607
NGA01000018-486-2	396	5	■■■■■■■■■■■■■■■■■■■■■■■■■■■■■■■■□□□□■■■■■■■	53	777777777760771	T1	3+	Kaduna	10.52001548	7.440000365
NGA01000018-590-1	443	5	■■■■■■■■■■■■■■■■■■■■■■■■■■■■■■■■□□□□■■■■■■■	53	777777777760771	T1	3+	Kaduna	10.52001548	7.440000365
NGA01000018-428-2	583	7	■■■■■■■■■■■■■■■■■■■■■■■■■■■■■■■■□□□□■■■■■■■	53	777777777760771	T1	3+	Kaduna	10.52001548	7.440000365
NGA01000019-1754-2	454	5	■■■■■■■■■■■■■■■■■■■■■■■■■■■■■■■■□□□□■■■■■■■	53	777777777760771	T1	2+	Kano	11.99997683	8.5200378
NGA01000020-234-2	614	8	■■■■■■■■■■■■■■■■■■■■■■■■■■■■■■■■□□□□■■■■■■■	53	777777777760771	T1	2+	Katsina	12.99040733	7.599990599
NGA01000020-093-2	615	8	■■■■■■■■■■■■■■■■■■■■■■■■■■■■■■■■□□□□■■■■■■■	53	777777777760771	T1	2+	Katsina	12.99040733	7.599990599
NGA01000020-255-2	622	8	■■■■■■■■■■■■■■■■■■■■■■■■■■■■■■■■□□□□■■■■■■■	53	777777777760771	T1	2+	Katsina	12.99040733	7.599990599
NGA01000020-257-1	630	8	■■■■■■■■■■■■■■■■■■■■■■■■■■■■■■■■□□□□■■■■■■■	53	777777777760771	T1	2+	Katsina	12.99040733	7.599990599
NGA01000020-089-1	631	8	■■■■■■■■■■■■■■■■■■■■■■■■■■■■■■■■□□□□■■■■■■■	53	777777777760771	T1	2+	Katsina	12.99040733	7.599990599
NGA01000020-267-3	646	8	■■■■■■■■■■■■■■■■■■■■■■■■■■■■■■■■□□□□■■■■■■■	53	777777777760771	T1	3+	Katsina	12.99040733	7.599990599
NGA01000020-099-3	647	8	■■■■■■■■■■■■■■■■■■■■■■■■■■■■■■■■□□□□■■■■■■■	53	777777777760771	T1	3+	Katsina	12.99040733	7.599990599
NGA01000022-026-1	393	5	■■■■■■■■■■■■■■■■■■■■■■■■■■■■■■■■□□□□■■■■■■■	53	777777777760771	T1	3+	Kogi	7.110404479	6.739939737
NGA01000023-775-2	480	6	■■■■■■■■■■■■■■■■■■■■■■■■■■■■■■■■□□□□■■■■■■■	53	777777777760771	T1	2+	Kwara	8.490010192	4.549995889
NGA01000023-453-1	527	6	■■■■■■■■■■■■■■■■■■■■■■■■■■■■■■■■□□□□■■■■■■■	53	777777777760771	T1	3+	Kwara	8.490010192	4.549995889
NGA01000024-1331-1	491	6	■■■■■■■■■■■■■■■■■■■■■■■■■■■■■■■■□□□□■■■■■■■	53	777777777760771	T1	3+	Lagos	6.443261653	3.391531071
NGA01000024-649-1	498	6	■■■■■■■■■■■■■■■■■■■■■■■■■■■■■■■■□□□□■■■■■■■	53	777777777760771	T1	2+	Lagos	6.443261653	3.391531071

NGA01000024-691-2	499	6	■■■■■■■■■■■■■■■■■■■■■■■■■■■■■■■■□□□□■■■■■■■	53	777777777760771	T1	2+	Lagos	6.443261653	3.391531071
NGA01000026-046-3	616	8	■■■■■■■■■■■■■■■■■■■■■■■■■■■■■■■■□□□□■■■■■■■	53	777777777760771	T1	2+	Niger	9.080413431	6.01001013
NGA010000026-154-1	617	8	■■■■■■■■■■■■■■■■■■■■■■■■■■■■■■■■□□□□■■■■■■■	53	777777777760771	T1	2+	Niger	9.080413431	6.01001013
NGA010000026-166-2	625	8	■■■■■■■■■■■■■■■■■■■■■■■■■■■■■■■■□□□□■■■■■■■	53	777777777760771	T1	3+	Niger	9.080413431	6.01001013
NGA01000026-544-3	632	8	■■■■■■■■■■■■■■■■■■■■■■■■■■■■■■■■□□□□■■■■■■■	53	777777777760771	T1	3+	Niger	9.080413431	6.01001013
NGA010000026-176-2	633	8	■■■■■■■■■■■■■■■■■■■■■■■■■■■■■■■■□□□□■■■■■■■	53	777777777760771	T1	3+	Niger	9.080413431	6.01001013
NGA010000026-1513-1	641	8	■■■■■■■■■■■■■■■■■■■■■■■■■■■■■■■■□□□□■■■■■■■	53	777777777760771	T1	3+	Niger	9.080413431	6.01001013
NGA01000026-184-2	648	8	■■■■■■■■■■■■■■■■■■■■■■■■■■■■■■■■□□□□■■■■■■■	53	777777777760771	T1	2+	Niger	9.080413431	6.01001013
NGA010000026-1515-3	649	8	■■■■■■■■■■■■■■■■■■■■■■■■■■■■■■■■□□□□■■■■■■■	53	777777777760771	T1	2+	Niger	9.080413431	6.01001013
NGA01000026-806-2	656	8	■■■■■■■■■■■■■■■■■■■■■■■■■■■■■■■■□□□□■■■■■■■	53	777777777760771	T1	2+	Niger	9.080413431	6.01001013
NGA01000027-781-3	605	7	■■■■■■■■■■■■■■■■■■■■■■■■■■■■■■■■□□□□■■■■■■■	53	777777777760771	T1	3+	Ogun	7.160427265	3.350017455
NGA010000028-009-3	618	8	■■■■■■■■■■■■■■■■■■■■■■■■■■■■■■■■□□□□■■■■■■■	53	777777777760771	T1	2+	Ondo	7.530430521	5.759999551
NGA010000028-008-1	634	8	■■■■■■■■■■■■■■■■■■■■■■■■■■■■■■■■□□□□■■■■■■■	53	777777777760771	T1	2+	Ondo	7.530430521	5.759999551
NGA010000028-029-1	642	8	■■■■■■■■■■■■■■■■■■■■■■■■■■■■■■■■□□□□■■■■■■■	53	777777777760771	T1	2+	Ondo	7.530430521	5.759999551
NGA010000028-047-2	650	8	■■■■■■■■■■■■■■■■■■■■■■■■■■■■■■■■□□□□■■■■■■■	53	777777777760771	T1	3+	Ondo	7.530430521	5.759999551
NGA01000029-163-1	566	7	■■■■■■■■■■■■■■■■■■■■■■■■■■■■■■■■□□□□■■■■■■■	53	777777777760771	T1	2+	Oyo	7.380026264	3.929982054
NGA01000029-234-2	606	7	■■■■■■■■■■■■■■■■■■■■■■■■■■■■■■■■□□□□■■■■■■■	53	777777777760771	T1	2+	Oyo	7.380026264	3.929982054
NGA010000033-004-3	774	9	■■■■■■■■■■■■■■■■■■■■■■■■■■■■■■■■□□□□■■■■■■■	53	777777777760771	T1	3+	Sokoto	13.06001548	5.240031289
NGA010000033-027-3	829	10	■■■■■■■■■■■■■■■■■■■■■■■■■■■■■■■■□□□□■■■■■■■	53	777777777760771	T1	2+	Sokoto	13.06001548	5.240031289
NGA010000036-498-2	889	10	■■■■■■■■■■■■■■■■■■■■■■■■■■■■■■■■□□□□■■■■■□■	278	777777777760761	T1	2+	Zamfara	12.1704057	6.659996296
NGA01000011-1612-2	188	3	■■■■■■■■■■■■■■■■■■■■■■■■■■■■■■■■□□□□■■■□■■■	52	777777777760731	T2	2+	Ebonyi	6.5218	8.1298
NGA01000015-1405-2	304	4	■■■■■■■■■■■■■■■■■■■■■■■■■■■■■■■■□□□□■■■□■■■	52	777777777760731	T2	3+	Gombe	10.29044293	11.16995357
NGA01000022-243-3	511	6	■■■■■■■■■■■■■■■■■■■■■■■■■■■■■■■■□□□□■■■□■■■	52	777777777760731	T2	2+	Kogi	7.110404479	6.739939737
NGA01000023-230-1	497	6	■■■■■■■■■■■■■■■■■■■■■■■■■■■■■■■■□□□□■■■□■■■	52	777777777760731	T2	2+	Kwara	8.490010192	4.549995889
NGA010000034-570-2	846	10	■■■■■■■■■■■■■■■■■■■■■■■■■■■■■■■■□□□□■■■□■■■	52	777777777760731	T2	3+	Taraba	8.900372741	11.36001949
NGA01000001-176-2	684	8	■■■■■■■■■■■■■■■■■■■■■■■■■■■■■■□■□□□□■■■■■■■	50	777777777720771	H3	3+	Abia	5.100397968	7.34998002
NGA01000001-045-1	698	8	■■■■■■■■■■■■■■■■■■■■■■■■■■■■■■□■□□□□■■■■■■■	50	777777777720771	H3	3+	Abia	5.100397968	7.34998002
NGA01000003-791-3	73	1	■■■■■■■■■■■■■■■■■■■■■■■■■■■■■■□■□□□□■■■■■■■	50	777777777720771	H3	3+	Anambra	6.210433572	7.06999711
NGA01000012-2559-2	206	3	■■■■■■■■■■■■■■■■■■■■■■■■■■■■■■□■□□□□■■■□■■■	49	777777777720731	H3	3+	Edo	6.340477314	5.620008096
NGA01000012-2196-1	237	3	■■■■■■■■■■■■■■■■■■■■■■■■■■■■■■□■□□□□■■■□■■■	49	777777777720731	H3	3+	Edo	6.340477314	5.620008096
NGA01000024-561-1	482	6	■■■■■■■■■■■■■■■■■■■■■■■■■■■■■■□■□□□□■■■□■■■	49	777777777720731	H3	2+	Lagos	6.443261653	3.391531071
NGA010000031-318-3	746	9	■■■■■■■■■■■■■■■■■■■■■■■■■■■■■■□■□□□□■■■□■■■	49	777777777720731	H3	3+	Plateau	9.929973978	8.890041055
NGA010000031-549-2	779	9	■■■■■■■■■■■■■■■■■■■■■■■■■■■■■■□■□□□□■■■□■■■	49	777777777720731	H3	2+	Plateau	9.929973978	8.890041055
NGA01000012-2269-2	253	3	■■■■■■■■■■■■■■■■■■■■■■■■■■■■■■□□□□□□□□■□■■■	836	777777777700131	U	3+	Edo	6.340477314	5.620008096
NGA010000035-1105-2	832	10	■■■■■■■■■■■■■■■■■■■■■■■■■■■■■□□□□□□□□□■□■■■	2660	777777777600131	Orphan	3+	Yobe	11.74899608	11.96600457
NGA01000006-601-2	108	2	■■■■■■■■■■■■■■■■■■■■■■■■■■■■□□□□■□■■■■■■■■■	236	777777777413771	EAI5	3+	Bayelsa	4.664030	6.036987
NGA010000035-896-3	855	10	■■■■■■■■■■■■■■■■■■■■■■■■■□□□■■■■□□□□■■■■■■■	2037	777777774360771	T1	2+	Yobe	11.74899608	11.96600457
NGA01000008-411-1	167	2	■■■■■■■■■■■■■■■■■■■■■■■■□□□□□□□■□□□□■■■■■■■	742	777777770020771	T2	3+	Borno	11.84996014	13.16001298
NGA01000017-184-2	578	7	■■■■■■■■■■■■■■■■■■■■■■■■□□□□□□□■□□□□■■■■■■■	742	777777770020771	T2	3+	Jigawa	11.7991891	9.350334607
NGA01000008-1254-3	176	2	■■■■■■■■■■■■■■■■■■■■■■■■□□□□□□□■□□□□■■■□■■■	316	777777770020731	T2	2+	Borno	11.84996014	13.16001298
NGA01000014-186-2	302	4	■■■■■■■■■■■■■■■■■■■■■■■■□□□□□□□□□□□□□□□□□□□	46	777777770000000	U (likely H)	2+	Ekiti	7.630372741	5.219980834
NGA010000033-009-3	909	11	■■■■■■■■■■■■■■■■■■■■■■■□■■■■■■□■□□□□■■■■■■■	631	777777767720771	H3	2+	Sokoto	13.06001548	5.240031289
NGA01000020-186-3	639	8	■■■■■■■■■■■■■■■■■■■■■■□□■■■■■■■■□□□□■■■■■■■	1580	777777747760771	T1	2+	Katsina	12.99040733	7.599990599
NGA01000026-591-2	640	8	■■■■■■■■■■■■■■■■■■■■■■□□■■■■■■■■□□□□■■■■■■■	1580	777777747760771	T1	3+	Niger	9.080413431	6.01001013
NGA01000001-494-1	659	8	■■■■■■■■■■■■■■■■■■■■■■□□□■■■■■■■□□□□■■■■■■■	61	777777743760771	CAM	3+	Abia	5.100397968	7.34998002
NGA01000001-492-2	667	8	■■■■■■■■■■■■■■■■■■■■■■□□□■■■■■■■□□□□■■■■■■■	61	777777743760771	CAM	3+	Abia	5.100397968	7.34998002
NGA01000001-175-3	676	8	■■■■■■■■■■■■■■■■■■■■■■□□□■■■■■■■□□□□■■■■■■■	61	777777743760771	CAM	3+	Abia	5.100397968	7.34998002
NGA01000002-346-2	78	1	■■■■■■■■■■■■■■■■■■■■■■□□□■■■■■■■□□□□■■■■■■■	61	777777743760771	CAM	2+	Adamawa	10.2703408	13.2700321
NGA01000002-213-2	560	7	■■■■■■■■■■■■■■■■■■■■■■□□□■■■■■■■□□□□■■■■■■■	61	777777743760771	CAM	2+	Adamawa	10.2703408	13.2700321
NGA01000004-628-2	92	1	■■■■■■■■■■■■■■■■■■■■■■□□□■■■■■■■□□□□■■■■■■■	61	777777743760771	CAM	2+	Akwa Ibom	5.007996056	7.849998524
NGA01000005-635-3	99	2	■■■■■■■■■■■■■■■■■■■■■■□□□■■■■■■■□□□□■■■■■■■	61	777777743760771	CAM	3+	Bauchi	10.3103642	9.84000891
NGA01000006-809-2	101	2	■■■■■■■■■■■■■■■■■■■■■■□□□■■■■■■■□□□□■■■■■■■	61	777777743760771	CAM	3+	Bayelsa	4.664030	6.036987
NGA01000006-556-2	155	2	■■■■■■■■■■■■■■■■■■■■■■□□□■■■■■■■□□□□■■■■■■■	61	777777743760771	CAM	3+	Bayelsa	4.664030	6.036987
NGA01000006-700-2	156	2	■■■■■■■■■■■■■■■■■■■■■■□□□■■■■■■■□□□□■■■■■■■	61	777777743760771	CAM	3+	Bayelsa	4.664030	6.036987
NGA01000006-579-2	567	7	■■■■■■■■■■■■■■■■■■■■■■□□□■■■■■■■□□□□■■■■■■■	61	777777743760771	CAM	3+	Bayelsa	4.664030	6.036987
NGA01000006-803-2	568	7	■■■■■■■■■■■■■■■■■■■■■■□□□■■■■■■■□□□□■■■■■■■	61	777777743760771	CAM	3+	Bayelsa	4.664030	6.036987
NGA01000007-523-1	117	2	■■■■■■■■■■■■■■■■■■■■■■□□□■■■■■■■□□□□■■■■■■■	61	777777743760771	CAM	2+	Benue	7.729979064	8.530011351
NGA01000007-707-2	134	2	■■■■■■■■■■■■■■■■■■■■■■□□□■■■■■■■□□□□■■■■■■■	61	777777743760771	CAM	2+	Benue	7.729979064	8.530011351

NGA01000007-610-2	149	2	■■■■■■■■■■■■■■■■■■■■■■□□□■■■■■■■□□□□■■■■■■■	61	777777743760771	CAM	3+	Benue	7.729979064	8.530011351
NGA01000008-133-3	120	2	■■■■■■■■■■■■■■■■■■■■■■□□□■■■■■■■□□□□■■■■■■■	61	777777743760771	CAM	3+	Borno	11.84996014	13.16001298
NGA01000008-093-2	136	2	■■■■■■■■■■■■■■■■■■■■■■□□□■■■■■■■□□□□■■■■■■■	61	777777743760771	CAM	2+	Borno	11.84996014	13.16001298
NGA01000008-090-3	144	2	■■■■■■■■■■■■■■■■■■■■■■□□□■■■■■■■□□□□■■■■■■■	61	777777743760771	CAM	3+	Borno	11.84996014	13.16001298
NGA01000008-1156-3	168	2	■■■■■■■■■■■■■■■■■■■■■■□□□■■■■■■■□□□□■■■■■■■	61	777777743760771	CAM	2+	Borno	11.84996014	13.16001298
NGA01000009-3822-2	154	2	■■■■■■■■■■■■■■■■■■■■■■□□□■■■■■■■□□□□■■■■■■■	61	777777743760771	CAM	3+	Cross River	4.960406513	8.330023558
NGA01000009-3527-2	169	2	■■■■■■■■■■■■■■■■■■■■■■□□□■■■■■■■□□□□■■■■■■■	61	777777743760771	CAM	3+	Cross River	4.960406513	8.330023558
NGA01000010-1271-2	193	3	■■■■■■■■■■■■■■■■■■■■■■□□□■■■■■■■□□□□■■■■■■■	61	777777743760771	CAM	3+	Delta	5.519958922	5.759999551
NGA01000010-1028-2	194	3	■■■■■■■■■■■■■■■■■■■■■■□□□■■■■■■■□□□□■■■■■■■	61	777777743760771	CAM	2+	Delta	5.519958922	5.759999551
NGA01000010-1113-2	208	3	■■■■■■■■■■■■■■■■■■■■■■□□□■■■■■■■□□□□■■■■■■■	61	777777743760771	CAM	3+	Delta	5.519958922	5.759999551
NGA01000010-1118-1	216	3	■■■■■■■■■■■■■■■■■■■■■■□□□■■■■■■■□□□□■■■■■■■	61	777777743760771	CAM	2+	Delta	5.519958922	5.759999551
NGA01000010-1152-2	232	3	■■■■■■■■■■■■■■■■■■■■■■□□□■■■■■■■□□□□■■■■■■■	61	777777743760771	CAM	3+	Delta	5.519958922	5.759999551
NGA01000010-1172-2	248	3	■■■■■■■■■■■■■■■■■■■■■■□□□■■■■■■■□□□□■■■■■■■	61	777777743760771	CAM	3+	Delta	5.519958922	5.759999551
NGA01000010-1015-2	265	3	■■■■■■■■■■■■■■■■■■■■■■□□□■■■■■■■□□□□■■■■■■■	61	777777743760771	CAM	3+	Delta	5.519958922	5.759999551
NGA01000010-1230-2	570	7	■■■■■■■■■■■■■■■■■■■■■■□□□■■■■■■■□□□□■■■■■■■	61	777777743760771	CAM	2+	Delta	5.519958922	5.759999551
NGA01000011-2073-3	203	3	■■■■■■■■■■■■■■■■■■■■■■□□□■■■■■■■□□□□■■■■■■■	61	777777743760771	CAM	3+	Ebonyi	6.5218	8.1298
NGA01000011-1684-3	212	3	■■■■■■■■■■■■■■■■■■■■■■□□□■■■■■■■□□□□■■■■■■■	61	777777743760771	CAM	3+	Ebonyi	6.5218	8.1298
NGA01000011-1868-3	218	3	■■■■■■■■■■■■■■■■■■■■■■□□□■■■■■■■□□□□■■■■■■■	61	777777743760771	CAM	3+	Ebonyi	6.5218	8.1298
NGA01000011-2085-1	219	3	■■■■■■■■■■■■■■■■■■■■■■□□□■■■■■■■□□□□■■■■■■■	61	777777743760771	CAM	3+	Ebonyi	6.5218	8.1298
NGA01000011-1869-2	226	3	■■■■■■■■■■■■■■■■■■■■■■□□□■■■■■■■□□□□■■■■■■■	61	777777743760771	CAM	3+	Ebonyi	6.5218	8.1298
NGA01000011-2139-3	227	3	■■■■■■■■■■■■■■■■■■■■■■□□□■■■■■■■□□□□■■■■■■■	61	777777743760771	CAM	3+	Ebonyi	6.5218	8.1298
NGA01000011-2182-2	251	3	■■■■■■■■■■■■■■■■■■■■■■□□□■■■■■■■□□□□■■■■■■■	61	777777743760771	CAM	3+	Ebonyi	6.5218	8.1298
NGA01000011-1999-2	266	3	■■■■■■■■■■■■■■■■■■■■■■□□□■■■■■■■□□□□■■■■■■■	61	777777743760771	CAM	2+	Ebonyi	6.5218	8.1298
NGA01000011-2229-2	267	3	■■■■■■■■■■■■■■■■■■■■■■□□□■■■■■■■□□□□■■■■■■■	61	777777743760771	CAM	3+	Ebonyi	6.5218	8.1298
NGA01000012-2931-1	189	3	■■■■■■■■■■■■■■■■■■■■■■□□□■■■■■■■□□□□■■■■■■■	61	777777743760771	CAM	3+	Edo	6.340477314	5.620008096
NGA01000012-2522-3	190	3	■■■■■■■■■■■■■■■■■■■■■■□□□■■■■■■■□□□□■■■■■■■	61	777777743760771	CAM	3+	Edo	6.340477314	5.620008096
NGA01000012-3039-2	197	3	■■■■■■■■■■■■■■■■■■■■■■□□□■■■■■■■□□□□■■■■■■■	61	777777743760771	CAM	3+	Edo	6.340477314	5.620008096
NGA01000012-1803-1	220	3	■■■■■■■■■■■■■■■■■■■■■■□□□■■■■■■■□□□□■■■■■■■	61	777777743760771	CAM	2+	Edo	6.340477314	5.620008096
NGA01000012-3288-1	222	3	■■■■■■■■■■■■■■■■■■■■■■□□□■■■■■■■□□□□■■■■■■■	61	777777743760771	CAM	3+	Edo	6.340477314	5.620008096
NGA01000012-3132-1	228	3	■■■■■■■■■■■■■■■■■■■■■■□□□■■■■■■■□□□□■■■■■■■	61	777777743760771	CAM	3+	Edo	6.340477314	5.620008096
NGA01000012-2819-2	260	3	■■■■■■■■■■■■■■■■■■■■■■□□□■■■■■■■□□□□■■■■■■■	61	777777743760771	CAM	3+	Edo	6.340477314	5.620008096
NGA01000012-2904-2	268	3	■■■■■■■■■■■■■■■■■■■■■■□□□■■■■■■■□□□□■■■■■■■	61	777777743760771	CAM	3+	Edo	6.340477314	5.620008096
NGA01000012-2330-2	269	3	■■■■■■■■■■■■■■■■■■■■■■□□□■■■■■■■□□□□■■■■■■■	61	777777743760771	CAM	3+	Edo	6.340477314	5.620008096
NGA01000014-305-3	287	4	■■■■■■■■■■■■■■■■■■■■■■□□□■■■■■■■□□□□■■■■■■■	61	777777743760771	CAM	3+	Ekiti	7.630372741	5.219980834
NGA01000014-127-1	294	4	■■■■■■■■■■■■■■■■■■■■■■□□□■■■■■■■□□□□■■■■■■■	61	777777743760771	CAM	2+	Ekiti	7.630372741	5.219980834
NGA01000014-200-2	310	4	■■■■■■■■■■■■■■■■■■■■■■□□□■■■■■■■□□□□■■■■■■■	61	777777743760771	CAM	3+	Ekiti	7.630372741	5.219980834
NGA01000014-229-2	326	4	■■■■■■■■■■■■■■■■■■■■■■□□□■■■■■■■□□□□■■■■■■■	61	777777743760771	CAM	3+	Ekiti	7.630372741	5.219980834
NGA01000014-295-3	366	4	■■■■■■■■■■■■■■■■■■■■■■□□□■■■■■■■□□□□■■■■■■■	61	777777743760771	CAM	3+	Ekiti	7.630372741	5.219980834
NGA01000014-300-2	577	7	■■■■■■■■■■■■■■■■■■■■■■□□□■■■■■■■□□□□■■■■■■■	61	777777743760771	CAM	3+	Ekiti	7.630372741	5.219980834
NGA01000013-781-2	231	3	■■■■■■■■■■■■■■■■■■■■■■□□□■■■■■■■□□□□■■■■■■■	61	777777743760771	CAM	2+	Enugu	6.450031351	7.499996703
NGA01000013-786-1	239	3	■■■■■■■■■■■■■■■■■■■■■■□□□■■■■■■■□□□□■■■■■■■	61	777777743760771	CAM	2+	Enugu	6.450031351	7.499996703
NGA01000013-882-2	263	3	■■■■■■■■■■■■■■■■■■■■■■□□□■■■■■■■□□□□■■■■■■■	61	777777743760771	CAM	3+	Enugu	6.450031351	7.499996703
NGA01000013-591-2	271	3	■■■■■■■■■■■■■■■■■■■■■■□□□■■■■■■■□□□□■■■■■■■	61	777777743760771	CAM	3+	Enugu	6.450031351	7.499996703
NGA01000013-529-1	575	7	■■■■■■■■■■■■■■■■■■■■■■□□□■■■■■■■□□□□■■■■■■■	61	777777743760771	CAM	3+	Enugu	6.450031351	7.499996703
NGA010000037-420-3	875	10	■■■■■■■■■■■■■■■■■■■■■■□□□■■■■■■■□□□□■■■■■■■	61	777777743760771	CAM	3+	Federal Capital Territory	9.083333149	7.533328002
NGA010000037-405-3	891	10	■■■■■■■■■■■■■■■■■■■■■■□□□■■■■■■■□□□□■■■■■■■	61	777777743760771	CAM	2+	Federal Capital Territory	9.083333149	7.533328002
NGA010000037-586-2	892	11	■■■■■■■■■■■■■■■■■■■■■■□□□■■■■■■■□□□□■■■■■■■	61	777777743760771	CAM	3+	Federal Capital Territory	9.083333149	7.533328002
NGA010000037-314-3	905	11	■■■■■■■■■■■■■■■■■■■■■■□□□■■■■■■■□□□□■■■■■■■	61	777777743760771	CAM	2+	Federal Capital Territory	9.083333149	7.533328002
NGA010000037-025-2	906	11	■■■■■■■■■■■■■■■■■■■■■■□□□■■■■■■■□□□□■■■■■■■	61	777777743760771	CAM	2+	Federal Capital Territory	9.083333149	7.533328002
NGA010000037-380-2	913	11	■■■■■■■■■■■■■■■■■■■■■■□□□■■■■■■■□□□□■■■■■■■	61	777777743760771	CAM	2+	Federal Capital Territory	9.083333149	7.533328002
NGA010000037-216-1	914	11	■■■■■■■■■■■■■■■■■■■■■■□□□■■■■■■■□□□□■■■■■■■	61	777777743760771	CAM	3+	Federal Capital Territory	9.083333149	7.533328002
NGA010000037-404-2	916	11	■■■■■■■■■■■■■■■■■■■■■■□□□■■■■■■■□□□□■■■■■■■	61	777777743760771	CAM	3+	Federal Capital Territory	9.083333149	7.533328002
NGA010000037-474-2	920	11	■■■■■■■■■■■■■■■■■■■■■■□□□■■■■■■■□□□□■■■■■■■	61	777777743760771	CAM	3+	Federal Capital Territory	9.083333149	7.533328002
NGA010000037-568-1	926	11	■■■■■■■■■■■■■■■■■■■■■■□□□■■■■■■■□□□□■■■■■■■	61	777777743760771	CAM	3+	Federal Capital Territory	9.083333149	7.533328002
NGA010000037-570-2	928	11	■■■■■■■■■■■■■■■■■■■■■■□□□■■■■■■■□□□□■■■■■■■	61	777777743760771	CAM	3+	Federal Capital Territory	9.083333149	7.533328002
NGA01000015-1286-3	288	4	■■■■■■■■■■■■■■■■■■■■■■□□□■■■■■■■□□□□■■■■■■■	61	777777743760771	CAM	3+	Gombe	10.29044293	11.16995357
NGA01000015-1406-1	312	4	■■■■■■■■■■■■■■■■■■■■■■□□□■■■■■■■□□□□■■■■■■■	61	777777743760771	CAM	3+	Gombe	10.29044293	11.16995357
NGA01000015-1446-3	328	4	■■■■■■■■■■■■■■■■■■■■■■□□□■■■■■■■□□□□■■■■■■■	61	777777743760771	CAM	3+	Gombe	10.29044293	11.16995357

NGA01000015-1462-2	336	4	■■■■■■■■■■■■■■■■■■■■■■□□□■■■■■■■□□□□■■■■■■■	61	777777743760771	CAM	3+	Gombe	10.29044293	11.16995357
NGA01000015-1242-1	343	4	■■■■■■■■■■■■■■■■■■■■■■□□□■■■■■■■□□□□■■■■■■■	61	777777743760771	CAM	2+	Gombe	10.29044293	11.16995357
NGA01000015-1536-2	360	4	■■■■■■■■■■■■■■■■■■■■■■□□□■■■■■■■□□□□■■■■■■■	61	777777743760771	CAM	3+	Gombe	10.29044293	11.16995357
NGA01000015-1265-2	367	4	■■■■■■■■■■■■■■■■■■■■■■□□□■■■■■■■□□□□■■■■■■■	61	777777743760771	CAM	3+	Gombe	10.29044293	11.16995357
NGA01000016-1045-3	291	4	■■■■■■■■■■■■■■■■■■■■■■□□□■■■■■■■□□□□■■■■■■■	61	777777743760771	CAM	3+	Imo	5.492997053	7.026003588
NGA01000017-012-1	284	4	■■■■■■■■■■■■■■■■■■■■■■□□□■■■■■■■□□□□■■■■■■■	61	777777743760771	CAM	3+	Jigawa	11.7991891	9.350334607
NGA01000017-017-1	292	4	■■■■■■■■■■■■■■■■■■■■■■□□□■■■■■■■□□□□■■■■■■■	61	777777743760771	CAM	3+	Jigawa	11.7991891	9.350334607
NGA01000017-046-2	308	4	■■■■■■■■■■■■■■■■■■■■■■□□□■■■■■■■□□□□■■■■■■■	61	777777743760771	CAM	3+	Jigawa	11.7991891	9.350334607
NGA01000017-051-3	316	4	■■■■■■■■■■■■■■■■■■■■■■□□□■■■■■■■□□□□■■■■■■■	61	777777743760771	CAM	3+	Jigawa	11.7991891	9.350334607
NGA01000017-078-2	332	4	■■■■■■■■■■■■■■■■■■■■■■□□□■■■■■■■□□□□■■■■■■■	61	777777743760771	CAM	3+	Jigawa	11.7991891	9.350334607
NGA01000017-149-2	356	4	■■■■■■■■■■■■■■■■■■■■■■□□□■■■■■■■□□□□■■■■■■■	61	777777743760771	CAM	3+	Jigawa	11.7991891	9.350334607
NGA01000017-008-2	363	4	■■■■■■■■■■■■■■■■■■■■■■□□□■■■■■■■□□□□■■■■■■■	61	777777743760771	CAM	3+	Jigawa	11.7991891	9.350334607
NGA01000017-178-2	364	4	■■■■■■■■■■■■■■■■■■■■■■□□□■■■■■■■□□□□■■■■■■■	61	777777743760771	CAM	3+	Jigawa	11.7991891	9.350334607
NGA01000017-010-3	371	4	■■■■■■■■■■■■■■■■■■■■■■□□□■■■■■■■□□□□■■■■■■■	61	777777743760771	CAM	3+	Jigawa	11.7991891	9.350334607
NGA01000017-179-2	372	4	■■■■■■■■■■■■■■■■■■■■■■□□□■■■■■■■□□□□■■■■■■■	61	777777743760771	CAM	3+	Jigawa	11.7991891	9.350334607
NGA01000017-133-2	378	5	■■■■■■■■■■■■■■■■■■■■■■□□□■■■■■■■□□□□■■■■■■■	61	777777743760771	CAM	3+	Jigawa	11.7991891	9.350334607
NGA01000017-152-2	402	5	■■■■■■■■■■■■■■■■■■■■■■□□□■■■■■■■□□□□■■■■■■■	61	777777743760771	CAM	2+	Jigawa	11.7991891	9.350334607
NGA01000017-157-3	410	5	■■■■■■■■■■■■■■■■■■■■■■□□□■■■■■■■□□□□■■■■■■■	61	777777743760771	CAM	2+	Jigawa	11.7991891	9.350334607
NGA01000017-185-2	434	5	■■■■■■■■■■■■■■■■■■■■■■□□□■■■■■■■□□□□■■■■■■■	61	777777743760771	CAM	3+	Jigawa	11.7991891	9.350334607
NGA01000018-406-2	387	5	■■■■■■■■■■■■■■■■■■■■■■□□□■■■■■■■□□□□■■■■■■■	61	777777743760771	CAM	3+	Kaduna	10.52001548	7.440000365
NGA01000018-417-2	403	5	■■■■■■■■■■■■■■■■■■■■■■□□□■■■■■■■□□□□■■■■■■■	61	777777743760771	CAM	3+	Kaduna	10.52001548	7.440000365
NGA01000018-562-2	404	5	■■■■■■■■■■■■■■■■■■■■■■□□□■■■■■■■□□□□■■■■■■■	61	777777743760771	CAM	3+	Kaduna	10.52001548	7.440000365
NGA01000018-588-2	412	5	■■■■■■■■■■■■■■■■■■■■■■□□□■■■■■■■□□□□■■■■■■■	61	777777743760771	CAM	3+	Kaduna	10.52001548	7.440000365
NGA01000018-583-2	419	5	■■■■■■■■■■■■■■■■■■■■■■□□□■■■■■■■□□□□■■■■■■■	61	777777743760771	CAM	3+	Kaduna	10.52001548	7.440000365
NGA01000018-573-2	420	5	■■■■■■■■■■■■■■■■■■■■■■□□□■■■■■■■□□□□■■■■■■■	61	777777743760771	CAM	3+	Kaduna	10.52001548	7.440000365
NGA01000018-518-2	427	5	■■■■■■■■■■■■■■■■■■■■■■□□□■■■■■■■□□□□■■■■■■■	61	777777743760771	CAM	3+	Kaduna	10.52001548	7.440000365
NGA01000018-590-1	428	5	■■■■■■■■■■■■■■■■■■■■■■□□□■■■■■■■□□□□■■■■■■■	61	777777743760771	CAM	2+	Kaduna	10.52001548	7.440000365
NGA01000018-485-1	435	5	■■■■■■■■■■■■■■■■■■■■■■□□□■■■■■■■□□□□■■■■■■■	61	777777743760771	CAM	3+	Kaduna	10.52001548	7.440000365
NGA01000018-555-1	444	5	■■■■■■■■■■■■■■■■■■■■■■□□□■■■■■■■□□□□■■■■■■■	61	777777743760771	CAM	3+	Kaduna	10.52001548	7.440000365
NGA01000019-3470-2	373	5	■■■■■■■■■■■■■■■■■■■■■■□□□■■■■■■■□□□□■■■■■■■	61	777777743760771	CAM	2+	Kano	11.99997683	8.5200378
NGA01000019-958-1	382	5	■■■■■■■■■■■■■■■■■■■■■■□□□■■■■■■■□□□□■■■■■■■	61	777777743760771	CAM	3+	Kano	11.99997683	8.5200378
NGA01000019-934-2	390	5	■■■■■■■■■■■■■■■■■■■■■■□□□■■■■■■■□□□□■■■■■■■	61	777777743760771	CAM	2+	Kano	11.99997683	8.5200378
NGA01000019-3718-2	413	5	■■■■■■■■■■■■■■■■■■■■■■□□□■■■■■■■□□□□■■■■■■■	61	777777743760771	CAM	3+	Kano	11.99997683	8.5200378
NGA01000019-801-2	414	5	■■■■■■■■■■■■■■■■■■■■■■□□□■■■■■■■□□□□■■■■■■■	61	777777743760771	CAM	2+	Kano	11.99997683	8.5200378
NGA01000019-3733-2	421	5	■■■■■■■■■■■■■■■■■■■■■■□□□■■■■■■■□□□□■■■■■■■	61	777777743760771	CAM	2+	Kano	11.99997683	8.5200378
NGA01000019-663-1	438	5	■■■■■■■■■■■■■■■■■■■■■■□□□■■■■■■■□□□□■■■■■■■	61	777777743760771	CAM	3+	Kano	11.99997683	8.5200378
NGA01000019-1113-2	445	5	■■■■■■■■■■■■■■■■■■■■■■□□□■■■■■■■□□□□■■■■■■■	61	777777743760771	CAM	3+	Kano	11.99997683	8.5200378
NGA01000019-1098-3	453	5	■■■■■■■■■■■■■■■■■■■■■■□□□■■■■■■■□□□□■■■■■■■	61	777777743760771	CAM	2+	Kano	11.99997683	8.5200378
NGA01000019-3222-1	460	5	■■■■■■■■■■■■■■■■■■■■■■□□□■■■■■■■□□□□■■■■■■■	61	777777743760771	CAM	2+	Kano	11.99997683	8.5200378
NGA01000020-039-1	662	8	■■■■■■■■■■■■■■■■■■■■■■□□□■■■■■■■□□□□■■■■■■■	61	777777743760771	CAM	2+	Katsina	12.99040733	7.599990599
NGA01000020-050-2	669	8	■■■■■■■■■■■■■■■■■■■■■■□□□■■■■■■■□□□□■■■■■■■	61	777777743760771	CAM	3+	Katsina	12.99040733	7.599990599
NGA01000020-122-1	671	8	■■■■■■■■■■■■■■■■■■■■■■□□□■■■■■■■□□□□■■■■■■■	61	777777743760771	CAM	3+	Katsina	12.99040733	7.599990599
NGA01000020-1312-2	678	8	■■■■■■■■■■■■■■■■■■■■■■□□□■■■■■■■□□□□■■■■■■■	61	777777743760771	CAM	2+	Katsina	12.99040733	7.599990599
NGA01000020-107-3	685	8	■■■■■■■■■■■■■■■■■■■■■■□□□■■■■■■■□□□□■■■■■■■	61	777777743760771	CAM	3+	Katsina	12.99040733	7.599990599
NGA01000020-3021-2	686	8	■■■■■■■■■■■■■■■■■■■■■■□□□■■■■■■■□□□□■■■■■■■	61	777777743760771	CAM	2+	Katsina	12.99040733	7.599990599
NGA01000020-3024-3	693	8	■■■■■■■■■■■■■■■■■■■■■■□□□■■■■■■■□□□□■■■■■■■	61	777777743760771	CAM	2+	Katsina	12.99040733	7.599990599
NGA01000020-3045-2	701	8	■■■■■■■■■■■■■■■■■■■■■■□□□■■■■■■■□□□□■■■■■■■	61	777777743760771	CAM	2+	Katsina	12.99040733	7.599990599
NGA01000021-074-2	391	5	■■■■■■■■■■■■■■■■■■■■■■□□□■■■■■■■□□□□■■■■■■■	61	777777743760771	CAM	2+	Kebbi	12.45041445	4.199939737
NGA01000021-493-3	408	5	■■■■■■■■■■■■■■■■■■■■■■□□□■■■■■■■□□□□■■■■■■■	61	777777743760771	CAM	2+	Kebbi	12.45041445	4.199939737
NGA010000021-323-2	915	11	■■■■■■■■■■■■■■■■■■■■■■□□□■■■■■■■□□□□■■■■■■■	61	777777743760771	CAM	2+	Kebbi	12.45041445	4.199939737
NGA010000021-258-1	925	11	■■■■■■■■■■■■■■■■■■■■■■□□□■■■■■■■□□□□■■■■■■■	61	777777743760771	CAM	3+	Kebbi	12.45041445	4.199939737
NGA01000022-013-2	385	5	■■■■■■■■■■■■■■■■■■■■■■□□□■■■■■■■□□□□■■■■■■■	61	777777743760771	CAM	3+	Kogi	7.110404479	6.739939737
NGA01000019-3714-2	397	5	■■■■■■■■■■■■■■■■■■■■■■□□□■■■■■■■□□□□■■■■■■■	61	777777743760771	CAM	2+	Kogi	7.110404479	6.739939737
NGA01000022-306-1	471	6	■■■■■■■■■■■■■■■■■■■■■■□□□■■■■■■■□□□□■■■■■■■	61	777777743760771	CAM	2+	Kogi	7.110404479	6.739939737
NGA01000022-282-1	479	6	■■■■■■■■■■■■■■■■■■■■■■□□□■■■■■■■□□□□■■■■■■■	61	777777743760771	CAM	2+	Kogi	7.110404479	6.739939737
NGA01000022-231-2	503	6	■■■■■■■■■■■■■■■■■■■■■■□□□■■■■■■■□□□□■■■■■■■	61	777777743760771	CAM	3+	Kogi	7.110404479	6.739939737
NGA01000022-276-2	519	6	■■■■■■■■■■■■■■■■■■■■■■□□□■■■■■■■□□□□■■■■■■■	61	777777743760771	CAM	2+	Kogi	7.110404479	6.739939737
NGA01000023-160-2	481	6	■■■■■■■■■■■■■■■■■■■■■■□□□■■■■■■■□□□□■■■■■■■	61	777777743760771	CAM	2+	Kwara	8.490010192	4.549995889

NGA01000023-230-1	489	6	■■■■■■■■■■■■■■■■■■■■■■□□□■■■■■■■□□□□■■■■■■■	61	777777743760771	CAM	2+	Kwara	8.490010192	4.549995889
NGA01000023-065-2	496	6	■■■■■■■■■■■■■■■■■■■■■■□□□■■■■■■■□□□□■■■■■■■	61	777777743760771	CAM	3+	Kwara	8.490010192	4.549995889
NGA01000023-056-1	504	6	■■■■■■■■■■■■■■■■■■■■■■□□□■■■■■■■□□□□■■■■■■■	61	777777743760771	CAM	2+	Kwara	8.490010192	4.549995889
NGA01000023-009-1	505	6	■■■■■■■■■■■■■■■■■■■■■■□□□■■■■■■■□□□□■■■■■■■	61	777777743760771	CAM	3+	Kwara	8.490010192	4.549995889
NGA01000023-064-2	512	6	■■■■■■■■■■■■■■■■■■■■■■□□□■■■■■■■□□□□■■■■■■■	61	777777743760771	CAM	2+	Kwara	8.490010192	4.549995889
NGA01000023-649-2	513	6	■■■■■■■■■■■■■■■■■■■■■■□□□■■■■■■■□□□□■■■■■■■	61	777777743760771	CAM	3+	Kwara	8.490010192	4.549995889
NGA01000023-082-3	521	6	■■■■■■■■■■■■■■■■■■■■■■□□□■■■■■■■□□□□■■■■■■■	61	777777743760771	CAM	3+	Kwara	8.490010192	4.549995889
NGA01000023-548-2	543	6	■■■■■■■■■■■■■■■■■■■■■■□□□■■■■■■■□□□□■■■■■■■	61	777777743760771	CAM	2+	Kwara	8.490010192	4.549995889
NGA01000023-803-1	544	6	■■■■■■■■■■■■■■■■■■■■■■□□□■■■■■■■□□□□■■■■■■■	61	777777743760771	CAM	2+	Kwara	8.490010192	4.549995889
NGA01000023-721-1	585	7	■■■■■■■■■■■■■■■■■■■■■■□□□■■■■■■■□□□□■■■■■■■	61	777777743760771	CAM	3+	kwara	8.490010192	4.549995889
NGA01000024-1240-1	483	6	■■■■■■■■■■■■■■■■■■■■■■□□□■■■■■■■□□□□■■■■■■■	61	777777743760771	CAM	2+	Lagos	6.443261653	3.391531071
NGA01000024-697-2	506	6	■■■■■■■■■■■■■■■■■■■■■■□□□■■■■■■■□□□□■■■■■■■	61	777777743760771	CAM	2+	Lagos	6.443261653	3.391531071
NGA01000024-1387-2	507	6	■■■■■■■■■■■■■■■■■■■■■■□□□■■■■■■■□□□□■■■■■■■	61	777777743760771	CAM	2+	Lagos	6.443261653	3.391531071
NGA01000024-702-1	514	6	■■■■■■■■■■■■■■■■■■■■■■□□□■■■■■■■□□□□■■■■■■■	61	777777743760771	CAM	3+	Lagos	6.443261653	3.391531071
NGA01000024-911-2	530	6	■■■■■■■■■■■■■■■■■■■■■■□□□■■■■■■■□□□□■■■■■■■	61	777777743760771	CAM	3+	Lagos	6.443261653	3.391531071
NGA01000024-940-2	531	6	■■■■■■■■■■■■■■■■■■■■■■□□□■■■■■■■□□□□■■■■■■■	61	777777743760771	CAM	2+	Lagos	6.443261653	3.391531071
NGA01000024-121-1	553	6	■■■■■■■■■■■■■■■■■■■■■■□□□■■■■■■■□□□□■■■■■■■	61	777777743760771	CAM	2+	Lagos	6.443261653	3.391531071
NGA01000024-830-2	554	6	■■■■■■■■■■■■■■■■■■■■■■□□□■■■■■■■□□□□■■■■■■■	61	777777743760771	CAM	2+	Lagos	6.443261653	3.391531071
NGA01000025-778-2	493	6	■■■■■■■■■■■■■■■■■■■■■■□□□■■■■■■■□□□□■■■■■■■	61	777777743760771	CAM	2+	Nasarawa	8.490423603	8.5200378
NGA01000025-2253-1	533	6	■■■■■■■■■■■■■■■■■■■■■■□□□■■■■■■■□□□□■■■■■■■	61	777777743760771	CAM	3+	Nasarawa	8.490423603	8.5200378
NGA01000026-041-2	664	8	■■■■■■■■■■■■■■■■■■■■■■□□□■■■■■■■□□□□■■■■■■■	61	777777743760771	CAM	2+	Niger	9.080413431	6.01001013
NGA01000026-1226-2	679	8	■■■■■■■■■■■■■■■■■■■■■■□□□■■■■■■■□□□□■■■■■■■	61	777777743760771	CAM	2+	Niger	9.080413431	6.01001013
NGA010000026-192-2	688	8	■■■■■■■■■■■■■■■■■■■■■■□□□■■■■■■■□□□□■■■■■■■	61	777777743760771	CAM	3+	Niger	9.080413431	6.01001013
NGA010000026-202-2	695	8	■■■■■■■■■■■■■■■■■■■■■■□□□■■■■■■■□□□□■■■■■■■	61	777777743760771	CAM	2+	Niger	9.080413431	6.01001013
NGA01000026-036-2	702	8	■■■■■■■■■■■■■■■■■■■■■■□□□■■■■■■■□□□□■■■■■■■	61	777777743760771	CAM	2+	Niger	9.080413431	6.01001013
NGA01000027-324-2	494	6	■■■■■■■■■■■■■■■■■■■■■■□□□■■■■■■■□□□□■■■■■■■	61	777777743760771	CAM	3+	Ogun	7.160427265	3.350017455
NGA01000027-439-2	518	6	■■■■■■■■■■■■■■■■■■■■■■□□□■■■■■■■□□□□■■■■■■■	61	777777743760771	CAM	3+	Ogun	7.160427265	3.350017455
NGA01000027-272-3	564	7	■■■■■■■■■■■■■■■■■■■■■■□□□■■■■■■■□□□□■■■■■■■	61	777777743760771	CAM	3+	Ogun	7.160427265	3.350017455
NGA01000027-316-2	572	7	■■■■■■■■■■■■■■■■■■■■■■□□□■■■■■■■□□□□■■■■■■■	61	777777743760771	CAM	3+	Ogun	7.160427265	3.350017455
NGA01000027-558	593	7	■■■■■■■■■■■■■■■■■■■■■■□□□■■■■■■■□□□□■■■■■■■	61	777777743760771	CAM	3+	Ogun	7.160427265	3.350017455
NGA01000027-570-3	596	7	■■■■■■■■■■■■■■■■■■■■■■□□□■■■■■■■□□□□■■■■■■■	61	777777743760771	CAM	3+	Ogun	7.160427265	3.350017455
NGA01000027-635-3	602	7	■■■■■■■■■■■■■■■■■■■■■■□□□■■■■■■■□□□□■■■■■■■	61	777777743760771	CAM	3+	Ogun	7.160427265	3.350017455
NGA01000027-787-2	608	7	■■■■■■■■■■■■■■■■■■■■■■□□□■■■■■■■□□□□■■■■■■■	61	777777743760771	CAM	3+	Ogun	7.160427265	3.350017455
NGA01000027-807-3	611	7	■■■■■■■■■■■■■■■■■■■■■■□□□■■■■■■■□□□□■■■■■■■	61	777777743760771	CAM	3+	Ogun	7.160427265	3.350017455
NGA010000028-006-2	743	9	■■■■■■■■■■■■■■■■■■■■■■□□□■■■■■■■□□□□■■■■■■■	61	777777743760771	CAM	2+	Ondo	7.530430521	5.759999551
NGA010000028-025-3	775	9	■■■■■■■■■■■■■■■■■■■■■■□□□■■■■■■■□□□□■■■■■■■	61	777777743760771	CAM	2+	Ondo	7.530430521	5.759999551
NGA010000028-014-3	902	11	■■■■■■■■■■■■■■■■■■■■■■□□□■■■■■■■□□□□■■■■■■■	61	777777743760771	CAM	2+	Ondo	7.530430521	5.759999551
NGA010000028-019-2	904	11	■■■■■■■■■■■■■■■■■■■■■■□□□■■■■■■■□□□□■■■■■■■	61	777777743760771	CAM	2+	Ondo	7.530430521	5.759999551
NGA010000030-087-1	721	9	■■■■■■■■■■■■■■■■■■■■■■□□□■■■■■■■□□□□■■■■■■■	61	777777743760771	CAM	2+	Osun	7.480433572	4.560021117
NGA010000030-112-1	737	9	■■■■■■■■■■■■■■■■■■■■■■□□□■■■■■■■□□□□■■■■■■■	61	777777743760771	CAM	2+	Osun	7.480433572	4.560021117
NGA010000030-047-1	744	9	■■■■■■■■■■■■■■■■■■■■■■□□□■■■■■■■□□□□■■■■■■■	61	777777743760771	CAM	3+	Osun	7.480433572	4.560021117
NGA010000030-113-2	745	9	■■■■■■■■■■■■■■■■■■■■■■□□□■■■■■■■□□□□■■■■■■■	61	777777743760771	CAM	2+	Osun	7.480433572	4.560021117
NGA010000030-121-3	753	9	■■■■■■■■■■■■■■■■■■■■■■□□□■■■■■■■□□□□■■■■■■■	61	777777743760771	CAM	3+	Osun	7.480433572	4.560021117
NGA010000030-051-1	760	9	■■■■■■■■■■■■■■■■■■■■■■□□□■■■■■■■□□□□■■■■■■■	61	777777743760771	CAM	3+	Osun	7.480433572	4.560021117
NGA010000030-052-3	768	9	■■■■■■■■■■■■■■■■■■■■■■□□□■■■■■■■□□□□■■■■■■■	61	777777743760771	CAM	3+	Osun	7.480433572	4.560021117
NGA010000030-052-3	776	9	■■■■■■■■■■■■■■■■■■■■■■□□□■■■■■■■□□□□■■■■■■■	61	777777743760771	CAM	3+	Osun	7.480433572	4.560021117
NGA010000033-006-3	790	9	■■■■■■■■■■■■■■■■■■■■■■□□□■■■■■■■□□□□■■■■■■■	61	777777743760771	CAM	3+	Osun	7.480433572	4.560021117
NGA010000030-279-2	793	9	■■■■■■■■■■■■■■■■■■■■■■□□□■■■■■■■□□□□■■■■■■■	61	777777743760771	CAM	3+	Osun	7.480433572	4.560021117
NGA01000029-233-1	603	7	■■■■■■■■■■■■■■■■■■■■■■□□□■■■■■■■□□□□■■■■■■■	61	777777743760771	CAM	3+	Oyo	7.380026264	3.929982054
NGA010000031-616-1	707	9	■■■■■■■■■■■■■■■■■■■■■■□□□■■■■■■■□□□□■■■■■■■	61	777777743760771	CAM	3+	Plateau	9.929973978	8.890041055
NGA010000031-621-2	708	9	■■■■■■■■■■■■■■■■■■■■■■□□□■■■■■■■□□□□■■■■■■■	61	777777743760771	CAM	3+	Plateau	9.929973978	8.890041055
NGA010000031-269-1	723	9	■■■■■■■■■■■■■■■■■■■■■■□□□■■■■■■■□□□□■■■■■■■	61	777777743760771	CAM	2+	Plateau	9.929973978	8.890041055
NGA010000031-049-1	731	9	■■■■■■■■■■■■■■■■■■■■■■□□□■■■■■■■□□□□■■■■■■■	61	777777743760771	CAM	2+	Plateau	9.929973978	8.890041055
NGA010000031-498-2	739	9	■■■■■■■■■■■■■■■■■■■■■■□□□■■■■■■■□□□□■■■■■■■	61	777777743760771	CAM	2+	Plateau	9.929973978	8.890041055
NGA010000031-552-3	787	9	■■■■■■■■■■■■■■■■■■■■■■□□□■■■■■■■□□□□■■■■■■■	61	777777743760771	CAM	3+	Plateau	9.929973978	8.890041055
NGA010000032-631-2	717	9	■■■■■■■■■■■■■■■■■■■■■■□□□■■■■■■■□□□□■■■■■■■	61	777777743760771	CAM	3+	Rivers	4.810002257	7.010000772
NGA010000032-965-2	718	9	■■■■■■■■■■■■■■■■■■■■■■□□□■■■■■■■□□□□■■■■■■■	61	777777743760771	CAM	2+	Rivers	4.810002257	7.010000772
NGA010000032-636-3	725	9	■■■■■■■■■■■■■■■■■■■■■■□□□■■■■■■■□□□□■■■■■■■	61	777777743760771	CAM	2+	Rivers	4.810002257	7.010000772

NGA010000032-647-2	741	9	■■■■■■■■■■■■■■■■■■■■■■□□□■■■■■■■□□□□■■■■■■■	61	777777743760771	CAM	2+	Rivers	4.810002257	7.010000772
NGA010000032-757-1	749	9	■■■■■■■■■■■■■■■■■■■■■■□□□■■■■■■■□□□□■■■■■■■	61	777777743760771	CAM	3+	Rivers	4.810002257	7.010000772
NGA010000032-452-2	756	9	■■■■■■■■■■■■■■■■■■■■■■□□□■■■■■■■□□□□■■■■■■■	61	777777743760771	CAM	2+	Rivers	4.810002257	7.010000772
NGA010000032-851-2	789	9	■■■■■■■■■■■■■■■■■■■■■■□□□■■■■■■■□□□□■■■■■■■	61	777777743760771	CAM	3+	Rivers	4.810002257	7.010000772
NGA010000033-001-3	750	9	■■■■■■■■■■■■■■■■■■■■■■□□□■■■■■■■□□□□■■■■■■■	61	777777743760771	CAM	3+	Sokoto	13.06001548	5.240031289
NGA010000033-002-3	758	9	■■■■■■■■■■■■■■■■■■■■■■□□□■■■■■■■□□□□■■■■■■■	61	777777743760771	CAM	3+	Sokoto	13.06001548	5.240031289
NGA010000033-003-3	766	9	■■■■■■■■■■■■■■■■■■■■■■□□□■■■■■■■□□□□■■■■■■■	61	777777743760771	CAM	3+	Sokoto	13.06001548	5.240031289
NGA010000033-010-3	804	10	■■■■■■■■■■■■■■■■■■■■■■□□□■■■■■■■□□□□■■■■■■■	61	777777743760771	CAM	2+	Sokoto	13.06001548	5.240031289
NGA010000033-012-3	820	10	■■■■■■■■■■■■■■■■■■■■■■□□□■■■■■■■□□□□■■■■■■■	61	777777743760771	CAM	3+	Sokoto	13.06001548	5.240031289
NGA010000033-013-3	828	10	■■■■■■■■■■■■■■■■■■■■■■□□□■■■■■■■□□□□■■■■■■■	61	777777743760771	CAM	2+	Sokoto	13.06001548	5.240031289
NGA010000033-014-3	836	10	■■■■■■■■■■■■■■■■■■■■■■□□□■■■■■■■□□□□■■■■■■■	61	777777743760771	CAM	3+	Sokoto	13.06001548	5.240031289
NGA010000033-026-3	837	10	■■■■■■■■■■■■■■■■■■■■■■□□□■■■■■■■□□□□■■■■■■■	61	777777743760771	CAM	3+	Sokoto	13.06001548	5.240031289
NGA010000033-015-3	844	10	■■■■■■■■■■■■■■■■■■■■■■□□□■■■■■■■□□□□■■■■■■■	61	777777743760771	CAM	3+	Sokoto	13.06001548	5.240031289
NGA010000033-016-3	852	10	■■■■■■■■■■■■■■■■■■■■■■□□□■■■■■■■□□□□■■■■■■■	61	777777743760771	CAM	3+	Sokoto	13.06001548	5.240031289
NGA010000033-019-3	868	10	■■■■■■■■■■■■■■■■■■■■■■□□□■■■■■■■□□□□■■■■■■■	61	777777743760771	CAM	2+	Sokoto	13.06001548	5.240031289
NGA010000033-020-3	876	10	■■■■■■■■■■■■■■■■■■■■■■□□□■■■■■■■□□□□■■■■■■■	61	777777743760771	CAM	3+	Sokoto	13.06001548	5.240031289
NGA010000033-021-3	884	10	■■■■■■■■■■■■■■■■■■■■■■□□□■■■■■■■□□□□■■■■■■■	61	777777743760771	CAM	3+	Sokoto	13.06001548	5.240031289
NGA010000034-954-3	799	10	■■■■■■■■■■■■■■■■■■■■■■□□□■■■■■■■□□□□■■■■■■■	61	777777743760771	CAM	3+	Taraba	8.900372741	11.36001949
NGA010000034-519-2	806	10	■■■■■■■■■■■■■■■■■■■■■■□□□■■■■■■■□□□□■■■■■■■	61	777777743760771	CAM	2+	Taraba	8.900372741	11.36001949
NGA010000034-1004-1	807	10	■■■■■■■■■■■■■■■■■■■■■■□□□■■■■■■■□□□□■■■■■■■	61	777777743760771	CAM	2+	Taraba	8.900372741	11.36001949
NGA010000034-544-3	830	10	■■■■■■■■■■■■■■■■■■■■■■□□□■■■■■■■□□□□■■■■■■■	61	777777743760771	CAM	3+	Taraba	8.900372741	11.36001949
NGA010000034-1128-3	831	10	■■■■■■■■■■■■■■■■■■■■■■□□□■■■■■■■□□□□■■■■■■■	61	777777743760771	CAM	2+	Taraba	8.900372741	11.36001949
NGA010000034-557-2	838	10	■■■■■■■■■■■■■■■■■■■■■■□□□■■■■■■■□□□□■■■■■■■	61	777777743760771	CAM	3+	Taraba	8.900372741	11.36001949
NGA010000034-789-2	839	10	■■■■■■■■■■■■■■■■■■■■■■□□□■■■■■■■□□□□■■■■■■■	61	777777743760771	CAM	2+	Taraba	8.900372741	11.36001949
NGA010000034-578-1	854	10	■■■■■■■■■■■■■■■■■■■■■■□□□■■■■■■■□□□□■■■■■■■	61	777777743760771	CAM	3+	Taraba	8.900372741	11.36001949
NGA010000034-469-1	861	10	■■■■■■■■■■■■■■■■■■■■■■□□□■■■■■■■□□□□■■■■■■■	61	777777743760771	CAM	2+	Taraba	8.900372741	11.36001949
NGA010000034-470-1	869	10	■■■■■■■■■■■■■■■■■■■■■■□□□■■■■■■■□□□□■■■■■■■	61	777777743760771	CAM	3+	Taraba	8.900372741	11.36001949
NGA010000034-623-1	870	10	■■■■■■■■■■■■■■■■■■■■■■□□□■■■■■■■□□□□■■■■■■■	61	777777743760771	CAM	3+	Taraba	8.900372741	11.36001949
NGA010000034-483-3	877	10	■■■■■■■■■■■■■■■■■■■■■■□□□■■■■■■■□□□□■■■■■■■	61	777777743760771	CAM	3+	Taraba	8.900372741	11.36001949
NGA010000034-504-1	885	10	■■■■■■■■■■■■■■■■■■■■■■□□□■■■■■■■□□□□■■■■■■■	61	777777743760771	CAM	3+	Taraba	8.900372741	11.36001949
NGA010000035-951-1	800	10	■■■■■■■■■■■■■■■■■■■■■■□□□■■■■■■■□□□□■■■■■■■	61	777777743760771	CAM	2+	Yobe	11.74899608	11.96600457
NGA010000035-1012-2	816	10	■■■■■■■■■■■■■■■■■■■■■■□□□■■■■■■■□□□□■■■■■■■	61	777777743760771	CAM	2+	Yobe	11.74899608	11.96600457
NGA010000035-166-2	840	10	■■■■■■■■■■■■■■■■■■■■■■□□□■■■■■■■□□□□■■■■■■■	61	777777743760771	CAM	2+	Yobe	11.74899608	11.96600457
NGA010000035-177-3	856	10	■■■■■■■■■■■■■■■■■■■■■■□□□■■■■■■■□□□□■■■■■■■	61	777777743760771	CAM	3+	Yobe	11.74899608	11.96600457
NGA010000035-929-1	863	10	■■■■■■■■■■■■■■■■■■■■■■□□□■■■■■■■□□□□■■■■■■■	61	777777743760771	CAM	2+	Yobe	11.74899608	11.96600457
NGA010000035-190-1	864	10	■■■■■■■■■■■■■■■■■■■■■■□□□■■■■■■■□□□□■■■■■■■	61	777777743760771	CAM	3+	Yobe	11.74899608	11.96600457
NGA010000035-933-1	871	10	■■■■■■■■■■■■■■■■■■■■■■□□□■■■■■■■□□□□■■■■■■■	61	777777743760771	CAM	2+	Yobe	11.74899608	11.96600457
NGA010000035-205-1	872	10	■■■■■■■■■■■■■■■■■■■■■■□□□■■■■■■■□□□□■■■■■■■	61	777777743760771	CAM	2+	Yobe	11.74899608	11.96600457
NGA010000035-211-3	880	10	■■■■■■■■■■■■■■■■■■■■■■□□□■■■■■■■□□□□■■■■■■■	61	777777743760771	CAM	2+	Yobe	11.74899608	11.96600457
NGA010000036-107-2	811	10	■■■■■■■■■■■■■■■■■■■■■■□□□■■■■■■■□□□□■■■■■■■	61	777777743760771	CAM	3+	Zamfara	12.1704057	6.659996296
NGA010000036-701-3	826	10	■■■■■■■■■■■■■■■■■■■■■■□□□■■■■■■■□□□□■■■■■■■	61	777777743760771	CAM	3+	Zamfara	12.1704057	6.659996296
NGA010000036-325-1	841	10	■■■■■■■■■■■■■■■■■■■■■■□□□■■■■■■■□□□□■■■■■■■	61	777777743760771	CAM	3+	Zamfara	12.1704057	6.659996296
NGA010000036-029-1	882	10	■■■■■■■■■■■■■■■■■■■■■■□□□■■■■■■■□□□□■■■■■■■	61	777777743760771	CAM	3+	Zamfara	12.1704057	6.659996296
NGA010000036-139-3	890	10	■■■■■■■■■■■■■■■■■■■■■■□□□■■■■■■■□□□□■■■■■■■	61	777777743760771	CAM	3+	Zamfara	12.1704057	6.659996296
NGA01000001-771-2	699	8	■■■■■■■■■■■■■■■■■■■■■■□□□■■■■■■■□□□□■■■■□■■	838	777777743760751	CAM	2+	Abia	5.100397968	7.34998002
NGA01000011-1919-2	242	3	■■■■■■■■■■■■■■■■■■■■■■□□□■■■■■■■□□□□■■■■□■■	838	777777743760751	CAM	3+	Ebonyi	6.5218	8.1298
NGA01000011-1960-2	258	3	■■■■■■■■■■■■■■■■■■■■■■□□□■■■■■■■□□□□■■■■□■■	838	777777743760751	CAM	3+	Ebonyi	6.5218	8.1298
NGA01000022-133-1	409	5	■■■■■■■■■■■■■■■■■■■■■■□□□■■■■■■■□□□□■■■■□■■	838	777777743760751	CAM	3+	Kogi	7.110404479	6.739939737
NGA01000022-251-3	495	6	■■■■■■■■■■■■■■■■■■■■■■□□□■■■■■■■□□□□■■■■□■■	838	777777743760751	CAM	3+	Kogi	7.110404479	6.739939737
NGA010000032-973-2	726	9	■■■■■■■■■■■■■■■■■■■■■■□□□■■■■■■■□□□□■■■■□■■	838	777777743760751	CAM	3+	Lagos	6.443261653	3.391531071
NGA01000008-121-3	96	2	■■■■■■■■■■■■■■■■■■■■■■□□□■■■■■■□□□□□■■■■■■■	NEW14*	777777743740771	CAM	3+	Borno	11.84996014	13.16001298
NGA01000012-2651-2	214	3	■■■■■■■■■■■■■■■■■■■■■■□□□■■■■■■□□□□□■■■■■■■	NEW14*	777777743740771	CAM	3+	Edo	6.340477314	5.620008096
NGA010000032-448-2	748	9	■■■■■■■■■■■■■■■■■■■■■■□□□■■■□□■■□□□□■■■■■■■	772	777777743460771	CAM	3+	Rivers	4.810002257	7.010000772
NGA01000008-1181-3	184	2	■■■■■■■■■■■■■■■■■■■■■■□□□□□□□■■■□□□□■■■□■■■	new-t	777777740160731	CAM	2+	Borno	11.84996014	13.16001298
NGA010000034-929-1	878	10	■■■■■■■■■■■■■■■■■■■■■□■■■■■■■■■■■□□□□■■■■■■■	86	777777737760771	T1	3+	Taraba	8.900372741	11.36001949
NGA01000001-481-1	636	8	■■■■■■■■■■■■■■■■■■■■□■■■■■■■■■■■■□□□□■■■■■■■	291	777777677760771	T1	3+	Abia	5.100397968	7.34998002
NGA01000010-1354-1	225	3	■■■■■■■■■■■■■■■■■■■■□■■■■■■■■■■■■□□□□■■■■■■■	291	777777677760771	T1	2+	Delta	5.519958922	5.759999551
NGA01000026-531-1	624	8	■■■■■■■■■■■■■■■■■■■■□■■■■■■■■■■■■□□□□■■■■■■■	291	777777677760771	T1	3+	Niger	9.080413431	6.01001013

NGA010000021-542-2	907	11	■■■■■■■■■■■■■■■■■■■■□■□□■■■■■■■■□□□□■■■■■■■	2298	777777647760771	T1	3+	Kebbi	12.45041445	4.199939737
NGA01000003-794-2	81	1	■■■■■■■■■■■■■■■■■■■■□□□□■■■■■■■■□□□□■■■■■■■	42	777777607760771	LAM9	2+	Anambra	6.210433572	7.06999711
NGA01000015-1583-3	297	4	■■■■■■■■■■■■■■■■■■■■□□□□■■■■■■■■□□□□■■■■■■■	42	777777607760771	LAM9	3+	Gombe	10.29044293	11.16995357
NGA01000021-316-2	416	5	■■■■■■■■■■■■■■■■■■■■□□□□■■■■■■■■□□□□■■■■■■■	42	777777607760771	LAM9	2+	Kebbi	12.45041445	4.199939737
NGA01000023-274-2	536	6	■■■■■■■■■■■■■■■■■■■■□□□□■■■■■■■□□□□□■■■■■■■	161	777777607740771	LAM9	3+	Kwara	8.490010192	4.549995889
NGA01000008-1019-3	160	2	■■■■■■■■■■■■■■■■■□■■■■■■■■■■■■■■□□□□■■■■■■■	119	777776777760771	X1	2+	Borno	11.84996014	13.16001298
NGA01000027-315-3	478	6	■■■■■■■■■■■■■■■■■□■■■■■■■■■■■■■■□□□□■■■■■■■	119	777776777760771	X1	3+	Ogun	7.160427265	3.350017455
NGA010000036-426-2	873	10	■■■■■■■■■■■■■■■■■□■■■■■■■■■■■■■■□□□□■■■■■■■	119	777776777760771	X1	2+	Zamfara	12.1704057	6.659996296
NGA01000012-2711-2	236	3	■■■■■■■■■■■■■■■□□□□■■■□□□■■■■■■■□□□□■■■■■■■	NEW13*	777770343760771	CAM	3+	Edo	6.340477314	5.620008096
NGA01000015-1229-2	335	4	■■■■■■■■■■■■■■■□□□□■■■□□□■■■■■■■□□□□■■■■■■■	NEW13*	777770343760771	CAM	3+	Gombe	10.29044293	11.16995357
NGA01000022-389-1	457	5	■■■■■■■■■■■■■■■□□□□■■■□□□■■■■■■■□□□□■■■■■■■	NEW13*	777770343760771	CAM	3+	Kogi	7.110404479	6.739939737
NGA01000024-1160-2	475	6	■■■■■■■■■■■■■■■□□□□■■■□□□■■■■■■■□□□□■■■■■■■	NEW13*	777770343760771	CAM	3+	Lagos	6.443261653	3.391531071
NGA010000026-1003-2	681	8	■■■■■■■■■■■■■■■□□□□■■■□□□■■■■■■■□□□□■■■■■■■	NEW13*	777770343760771	CAM	3+	Niger	9.080413431	6.01001013
NGA01000017-139-1	386	5	■■■■■■■■■■■■■■■□□□□□□□□□□□□□□□□□□□□□■■■■■■■	NEW12	777770000000771	CAM	2+	Jigawa	11.7991891	9.350334607
NGA01000021-475-1	392	5	■■■■■■■■■■■■■■■□□□□□□□□□□□□□□□□□□□□□■■■■■■■	NEW12	777770000000771	CAM	2+	Kebbi	12.45041445	4.199939737
NGA01000015-660-3	337	4	■■■■■■■■■■■■■■■□□□□□□□□□□□□□□□□□□□□□■■■□■■■	1204	777770000000731	CAM	3+	Gombe	10.29044293	11.16995357
NGA01000019-3713-3	405	5	■■■■■■■■■■■■■■■□□□□□□□□□□□□□□□□□□□□□■■■□■■■	1204	777770000000731	CAM	3+	Kano	11.99997683	8.5200378
NGA01000026-1561-3	687	8	■■■■■■■■■■■■■■■□□□□□□□□□□□□□□□□□□□□□■■■□■■■	1204	777770000000731	CAM	3+	Niger	9.080413431	6.01001013
NGA010000030-045-1	736	9	■■■■■■■■■■■■■□□□□□□□□□□■■■■■■■■■□□□□■■■■■■■	159	777740017760771	T1	2+	Osun	7.480433572	4.560021117
NGA01000017-019-2	442	5	■■■■■■■■■■■■□■■■■■■■■■■■■■■■■■■■□□□□■□□■■■■	157	777737777760471	T3	3+	Jigawa	11.7991891	9.350334607
NGA01000010-995-2	249	3	■■■■■■■■■■■■□■■■■■■■■□□□■■■■■■■□□□□■■■■■■■	2550	777737743760771	CAM	3+	Delta	5.519958922	5.759999551
NGA01000019-960-2	374	5	■■■■■■■■■■■■□■■■■■■■■□□□■■■■■■■□□□□■■■■■■■	2550	777737743760771	CAM	3+	Kano	11.99997683	8.5200378
NGA010000028-005-2	727	9	■■■■■■■■■■■■□■■■■■■■■□□□■■■■■■■□□□□■■■■■■■	2550	777737743760771	CAM	2+	Ondo	7.530430521	5.759999551
NGA01000018-484-2	436	5	■■■■■■■■■■■■□□■■■■■■■■□□□■■■■■■■□□□□■■■■■■■	1065	777717743760771	CAM	2+	Kaduna	10.52001548	7.440000365
NGA01000012-2212-3	221	3	■■■■■■■■■■■■□□□□□□□□□□□□□□□■■■■■□□□□■■■■■■■	2540	777700000760771	T1	3+	Edo	6.340477314	5.620008096
NGA01000014-026-1	303	4	■■■■■■■■■■■■□□□□□□□□□□□□□□□□□□□□□□□□□□□□□□□	1869	777700000000000	U	3+	Ekiti	7.630372741	5.219980834
NGA01000014-279-2	350	4	■■■■■■■■■■■■□□□□□□□□□□□□□□□□□□□□□□□□□□□□□□□	1869	777700000000000	U	3+	Ekiti	7.630372741	5.219980834
NGA010000021-300-1	921	11	■■■■■■■■■■□■■■■■■■■■■□□□■■■■■■■□□□□■■■■■■■	Orphan	777577743760771	CAM	2+	Kebbi	12.45041445	4.199939737
NGA010000030-274-2	777	9	■■■■■■■■■□■■■■□■■■■■■■□□□■■■■■■■□□□□■■□■■■■	Koro-Koro 2013	777367743760671	CAM	2+	Osun	7.480433572	4.560021117
NGA010000031-320-3	762	9	■■■■■■■■■□■■■■□■■■■■■■□□□■■■■■■■□□□□■■□■■■■	Koro-Koro 2013	777367743760671	CAM	2+	Plateau	9.929973978	8.890041055
NGA010000031-330-1	778	9	■■■■■■■■■□■■■■□■■■■■■■□□□■■■■■■■□□□□■■□■■■■	Koro-Koro 2013	777367743760671	CAM	3+	Plateau	9.929973978	8.890041055
NGA010000031-391-3	786	9	■■■■■■■■■□■■■■□■■■■■■■□□□■■■■■■■□□□□■■□■■■■	Koro-Koro 2013	777367743760671	CAM	3+	Plateau	9.929973978	8.890041055
NGA010000031-405-3	794	9	■■■■■■■■■□■■■■□■■■■■■■□□□■■■■■■■□□□□■■□■■■■	Koro-Koro 2013	777367743760671	CAM	3+	Plateau	9.929973978	8.890041055
NGA010000031-564-2	795	9	■■■■■■■■■□■■■■□■■■■■■■□□□■■■■■■■□□□□■■□■■■■	Koro-Koro 2013	777367743760671	CAM	2+	Plateau	9.929973978	8.890041055
NGA01000017-061-2	324	4	■■■■■■■■■□□□□□□■■■■■■■■■■■■■■■□■□□□□■■■■■■■	new-s	777007777720771	H	3+	Jigawa	11.7991891	9.350334607
NGA01000012-2720-1	244	3	■■■■■■■■□□■■■■■■■■■■■□□□□□□□□□□□□□□□□□□□□□■	NEW10	776377700000001	U	3+	Edo	6.340477314	5.620008096
NGA01000019-806-3	406	5	■■■■■■■■□□■■■■■■■■■■■□□□□□□□□□□□□□□□□□□□□□■	NEW10	776377700000001	U	3+	Kano	11.99997683	8.5200378
NGA01000009-4006-1	114	2	■■■■■■■■□□□□■■■■■■■■■■■■■■■■■■■■□□□□■■■■■■■	1223	776077777760771	T1	3+	Cross River	4.960406513	8.330023558
NGA01000009-3472-2	129	2	■■■■■■■■□□□□□□□■■□□□■□□□□□■□□□■■□□□□■□■■■■■	new	776006101060571	CAM	2+	Cross River	4.960406513	8.330023558
NGA010000021-412-2	929	11	■■■■■■■□□□■■■■■■■■■■■■■■■■■■■■■■□■■□■■■■■■■	Orphan	774377777766771	Orphan	3+	Kebbi	12.45041445	4.199939737
NGA01000004-165-2	11	1	■■■■■■■□□□□□■■■■■■■■■■■■■■■■■■■■■■■■□□□■■■■	438	774077777777071	Afri_2	2+	Akwa Ibom	5.007996056	7.849998524
NGA01000004-661-3	13	1	■■■■■■■□□□□□■■■■■■■■■■■■■■■■■■■■■■■■□□□■■■■	438	774077777777071	Afri_2	2+	Akwa Ibom	5.007996056	7.849998524
NGA01000003-659-1	25	1	■■■■■■■□□□□□■■■■■■■■■■■■■■■■■■■■■■■■□□□■■■■	438	774077777777071	Afri_2	3+	Anambra	6.210433572	7.06999711
NGA01000006-577-2	179	2	■■■■■■■□□□□□■■■■■■■■■■■■■■■■■■■■■■■■□□□■■■■	438	774077777777071	Afri_2	3+	Bayelsa	4.664030	6.036987
NGA01000011-2162-2	235	3	■■■■■■■□□□□□■■■■■■■■■■■■■■■■■■■■■■■■□□□■■■■	438	774077777777071	Afri_2	3+	Ebonyi	6.5218	8.1298
NGA01000011-2163-3	243	3	■■■■■■■□□□□□■■■■■■■■■■■■■■■■■■■■■■■■□□□■■■■	438	774077777777071	Afri_2	3+	Ebonyi	6.5218	8.1298
NGA01000013-596-2	279	3	■■■■■■■□□□□□■■■■■■■■■■■■■■■■■■■■■■■■□□□■■■■	438	774077777777071	Afri_2	3+	Enugu	6.450031351	7.499996703
NGA01000017-092-2	340	4	■■■■■■■□□□□□■■■■■■■■■■■■■■■■■■■■■■■■□□□■■■■	438	774077777777071	Afri_2	3+	Jigawa	11.7991891	9.350334607
NGA01000025-2255-3	541	6	■■■■■■■□□□□□■■■■■■■■■■■■■■■■■■■■■■■■□□□■■■■	438	774077777777071	Afri_2	3+	Nasarawa	8.490423603	8.5200378
NGA010000032-516-3	764	9	■■■■■■■□□□□□■■■■■■■■■■■■■■■■■■■■■■■■□□□■■■■	438	774077777777071	Afri_2	3+	Rivers	4.810002257	7.010000772
NGA010000032-765-1	765	9	■■■■■■■□□□□□■■■■■■■■■■■■■■■■■■■■■■■■□□□■■■■	438	774077777777071	Afri_2	2+	Rivers	4.810002257	7.010000772
NGA01000007-817-3	95	2	■■■■■■■□□□□□■■■■■■■■■■■■□□□□□□□□□■□■□□□■■■■	856	774077770005071	AFRI_3	3+	Benue	7.729979064	8.530011351
NGA01000007-831-2	111	2	■■■■■■■□□□□□■■■■■■■■■■■■□□□□□□□□□■□■□□□■■■■	856	774077770005071	AFRI_3	3+	Benue	7.729979064	8.530011351
NGA01000007-740-1	150	2	■■■■■■■□□□□□■■■■■■■■■■■■□□□□□□□□□■□■□□□■■■■	856	774077770005071	AFRI_3	3+	Benue	7.729979064	8.530011351
NGA01000007-743-1	158	2	■■■■■■■□□□□□■■■■■■■■■■■■□□□□□□□□□■□■□□□■■■■	856	774077770005071	AFRI_3	3+	Benue	7.729979064	8.530011351
NGA01000007-782-2	174	2	■■■■■■■□□□□□■■■■■■■■■■■■□□□□□□□□□■□■□□□■■■■	856	774077770005071	AFRI_3	3+	Benue	7.729979064	8.530011351
NGA01000007-805-2	182	2	■■■■■■■□□□□□■■■■■■■■■■■■□□□□□□□□□■□■□□□■■■■	856	774077770005071	AFRI_3	3+	Benue	7.729979064	8.530011351
NGA01000008-434-2	183	2	■■■■■■■□□□□□■■■■■■■■■■■■□□□□□□□□□■□■□□□■■■■	856	774077770005071	AFRI_3	3+	Borno	11.84996014	13.16001298

NGA01000001-134-3	668	8	■■■■■■■□□□□□■■■■■■■■□□□□■■■■■■■■■■■■□□□■■■■	331	774077607777071	Afri_2	2+	Abia	5.100397968	7.34998002
NGA01000001-452-2	900	11	■■■■■■■□□□□□■■■■■■■■□□□□■■■■■■■■■■■■□□□■■■■	331	774077607777071	Afri_2	2+	Abia	5.100397968	7.34998002
NGA01000004-600-2	12	1	■■■■■■■□□□□□■■■■■■■■□□□□■■■■■■■■■■■■□□□■■■■	331	774077607777071	Afri_2	2+	Akwa Ibom	5.007996056	7.849998524
NGA01000006-572-3	171	2	■■■■■■■□□□□□■■■■■■■■□□□□■■■■■■■■■■■■□□□■■■■	331	774077607777071	Afri_2	3+	Bayelsa	4.664030	6.036987
NGA01000007-672-2	157	2	■■■■■■■□□□□□■■■■■■■■□□□□■■■■■■■■■■■■□□□■■■■	331	774077607777071	Afri_2	2+	Benue	7.729979064	8.530011351
NGA01000009-2602-3	106	2	■■■■■■■□□□□□■■■■■■■■□□□□■■■■■■■■■■■■□□□■■■■	331	774077607777071	Afri_2	3+	Cross River	4.960406513	8.330023558
NGA01000009-3484-2	137	2	■■■■■■■□□□□□■■■■■■■■□□□□■■■■■■■■■■■■□□□■■■■	331	774077607777071	Afri_2	3+	Cross River	4.960406513	8.330023558
NGA01000009-3753-2	138	2	■■■■■■■□□□□□■■■■■■■■□□□□■■■■■■■■■■■■□□□■■■■	331	774077607777071	Afri_2	2+	Cross River	4.960406513	8.330023558
NGA01000012-3021-2	205	3	■■■■■■■□□□□□■■■■■■■■□□□□■■■■■■■■■■■■□□□■■■■	331	774077607777071	Afri_2	3+	Edo	6.340477314	5.620008096
NGA01000014-088-3	576	7	■■■■■■■□□□□□■■■■■■■■□□□□■■■■■■■■■■■■□□□■■■■	331	774077607777071	Afri_2	3+	Ekiti	7.630372741	5.219980834
NGA010000037-513-2	898	11	■■■■■■■□□□□□■■■■■■■■□□□□■■■■■■■■■■■■□□□■■■■	331	774077607777071	Afri_2	3+	Federal Capital Territory	9.083333149	7.533328002
NGA01000016-1286-1	339	4	■■■■■■■□□□□□■■■■■■■■□□□□■■■■■■■■■■■■□□□■■■■	331	774077607777071	Afri_2	2+	Imo	5.492997053	7.026003588
NGA01000016-1013-2	370	4	■■■■■■■□□□□□■■■■■■■■□□□□■■■■■■■■■■■■□□□■■■■	331	774077607777071	Afri_2	3+	Imo	5.492997053	7.026003588
NGA01000019-1172-3	437	5	■■■■■■■□□□□□■■■■■■■■□□□□■■■■■■■■■■■■□□□■■■■	331	774077607777071	Afri_2	3+	Kano	11.99997683	8.5200378
NGA01000022-281-2	487	6	■■■■■■■□□□□□■■■■■■■■□□□□■■■■■■■■■■■■□□□■■■■	331	774077607777071	Afri_2	2+	Kogi	7.110404479	6.739939737
NGA01000024-592-1	490	6	■■■■■■■□□□□□■■■■■■■■□□□□■■■■■■■■■■■■□□□■■■■	331	774077607777071	Afri_2	3+	Lagos	6.443261653	3.391531071
NGA010000032-999-1	742	9	■■■■■■■□□□□□■■■■■■■■□□□□■■■■■■■■■■■■□□□■■■■	331	774077607777071	Afri_2	3+	Rivers	4.810002257	7.010000772
NGA010000032-764-1	757	9	■■■■■■■□□□□□■■■■■■■■□□□□■■■■■■■■■■■■□□□■■■■	331	774077607777071	Afri_2	2+	Rivers	4.810002257	7.010000772
NGA010000032-839-2	773	9	■■■■■■■□□□□□■■■■■■■■□□□□■■■■■■■■■■■■□□□■■■■	331	774077607777071	Afri_2	2+	Rivers	4.810002257	7.010000772
NGA010000032-559-3	780	9	■■■■■■■□□□□□■■■■■■■■□□□□■■■■■■■■■■■■□□□■■■■	331	774077607777071	Afri_2	3+	Rivers	4.810002257	7.010000772
NGA010000032-583-2	796	9	■■■■■■■□□□□□■■■■■■■■□□□□■■■■■■■■■■■■□□□■■■■	331	774077607777071	Afri_2	3+	Rivers	4.810002257	7.010000772
NGA010000034-526-3	814	10	■■■■■■■□□□□□■■■■■■■■□□□□■■■■■■■■■■■■□□□■■■■	331	774077607777071	Afri_2	3+	Taraba	8.900372741	11.36001949
NGA010000036-715-2	834	10	■■■■■■■□□□□□■■■■■■■■□□□□■■■■■■■■■■■■□□□■■■■	331	774077607777071	Afri_2	2+	Zamfara	12.1704057	6.659996296
NGA01000009-3401-3	113	2	■■■■■■■□□□□□■■■■■■■■□□□□■■■■■■■■■■■■□□□■□□■	NEW9	774077607777041	Afri_2	2+	Cross River	4.960406513	8.330023558
NGA01000010-1163-2	240	3	■■■■■■■□□□□□■■■■■■■■□□□□■■■■■■■■■■■■□□□■□□■	NEW9	774077607777041	Afri_2	3+	Delta	5.519958922	5.759999551
NGA010000032-621-3	710	9	■■■■■■■□□□□□■■■■■■■■□□□□■■■■■■■■■■■■□□□□□□□	Orphan	774077607777000	AFRI_2 (?)	3+	Rivers	4.810002257	7.010000772
NGA01000010-1102-2	200	3	■■■■■■■□□□□□■■■■■■■■□□□□■■■■■■■□□□□□□□□■■■■	NEW6	774077607740071	Afri_2	2+	Delta	5.519958922	5.759999551
NGA01000010-1184-1	256	3	■■■■■■■□□□□□■■■■■■■■□□□□■■■■■■■□□□□□□□□■■■■	NEW6	774077607740071	Afri_2	2+	Delta	5.519958922	5.759999551
NGA01000006-800-1	180	2	■■■■■■■□□□□□■■■■■■■■□□□□■■□□□■■■■□□□□■■■	new-r	774077607617031	Afri_2	3+	Bayelsa	4.664030	6.036987
NGA01000011-2013-3	187	3	■■■■■■■□□□□□■■■■■■■■□□□□■■■■□□■■■■■■□□□■■■■	new-q	774077607477071	Afri_2	3+	Ebonyi	6.5218	8.1298
NGA010000032-444-3	740	9	■■■■■■■□□□□□■■■■■■■■□□□□■■□■■■■■■■■■□□□■■■■	Orphan	774077606777071	AFRI_2	3+	Rivers	4.810002257	7.010000772
NGA010000032-607-3	709	9	■■■■■■■□□□□□■■■■■■■■□□□□■□□□■■■■■■■■□□□■■■■	Orphan	774077604377071	AFRI_2	2+	Rivers	4.810002257	7.010000772
NGA01000012-2773-2	252	3	■■■■■■■□□□□□■■■■■■■■□□□□□□□□□□□□□□□□□□□■■■■	new-p	774077600000071	Afri_2	3+	Edo	6.340477314	5.620008096
NGA01000010-962-1	241	3	■■■■■■■□□□□□■■■■■■■■□□□□□□□□□□□□□□□□□□□□■■■	NEW11*	774077600000031	Afri_2	2+	Delta	5.519958922	5.759999551
NGA01000023-365-2	488	6	■■■■■■■□□□□□■■■■■■■■□□□□□□□□□□□□□□□□□□□□■■■	NEW11*	774077600000031	Afri_2	3+	Kwara	8.490010192	4.549995889
NGA010000031-545-3	763	9	■■■■■■■□□□□□■■■■■■■■□□□□□□□□□□□□□□□□□□□□■■■	NEW11*	774077600000031	Afri_2	3+	Plateau	9.929973978	8.890041055
NGA010000032-643-2	733	9	■■■■■■■□□□□□■■■■■■■■□□□□□□□□□□□□□□□□□□□□■■■	NEW11*	774077600000031	Afri_2	3+	Rivers	4.810002257	7.010000772
NGA010000032-847-2	781	9	■■■■■■■□□□□□■■■■■■■■□□□□□□□□□□□□□□□□□□□□■■■	NEW11*	774077600000031	Afri_2	2+	Rivers	4.810002257	7.010000772
NGA01000007-744-1	166	2	■■■■■■■□□□□□■■■■■■■□□□■■□□□□□□□□□■□■□□□■■■■	new-o	774077430005071	Afri_2	3+	Benue	7.729979064	8.530011351
NGA01000001-1000-2	691	8	■■■■■■■□□□□□■■■■■■□□□□□□■■■■■■■■■■■■□□□■■■■	761	774077007777071	AFRI_3	2+	Abia	5.100397968	7.34998002
NGA01000013-759-2	191	3	■■■■■■■□□□□□■□□□□□□□□□□□□□□□□□□□□□□□□□□□□□□	NEW5	774040000000000	U	2+	Enugu	6.450031351	7.499996703
NGA01000013-805-3	199	3	■■■■■■■□□□□□■□□□□□□□□□□□□□□□□□□□□□□□□□□□□□□	NEW5	774040000000000	U	2+	Enugu	6.450031351	7.499996703
NGA01000013-888-3	207	3	■■■■■■■□□□□□■□□□□□□□□□□□□□□□□□□□□□□□□□□□□□□	NEW5	774040000000000	U	2+	Enugu	6.450031351	7.499996703
NGA01000013-920-2	215	3	■■■■■■■□□□□□■□□□□□□□□□□□□□□□□□□□□□□□□□□□□□□	NEW5	774040000000000	U	2+	Enugu	6.450031351	7.499996703
NGA01000013-961-2	223	3	■■■■■■■□□□□□■□□□□□□□□□□□□□□□□□□□□□□□□□□□□□□	NEW5	774040000000000	U	2+	Enugu	6.450031351	7.499996703
NGA01000013-002-2	230	3	■■■■■■■□□□□□■□□□□□□□□□□□□□□□□□□□□□□□□□□□□□□	NEW5	774040000000000	U	2+	Enugu	6.450031351	7.499996703
NGA01000013-032-2	238	3	■■■■■■■□□□□□■□□□□□□□□□□□□□□□□□□□□□□□□□□□□□□	NEW5	774040000000000	U	2+	Enugu	6.450031351	7.499996703
NGA01000013-093-1	246	3	■■■■■■■□□□□□■□□□□□□□□□□□□□□□□□□□□□□□□□□□□□□	NEW5	774040000000000	U	2+	Enugu	6.450031351	7.499996703
NGA01000013-179-3	254	3	■■■■■■■□□□□□■□□□□□□□□□□□□□□□□□□□□□□□□□□□□□□	NEW5	774040000000000	U	2+	Enugu	6.450031351	7.499996703
NGA01000013-421-1	262	3	■■■■■■■□□□□□■□□□□□□□□□□□□□□□□□□□□□□□□□□□□□□	NEW5	774040000000000	U	2+	Enugu	6.450031351	7.499996703
NGA01000013-451-1	270	3	■■■■■■■□□□□□■□□□□□□□□□□□□□□□□□□□□□□□□□□□□□□	NEW5	774040000000000	U	2+	Enugu	6.450031351	7.499996703
NGA01000013-708-2	278	3	■■■■■■■□□□□□■□□□□□□□□□□□□□□□□□□□□□□□□□□□□□□	NEW5	774040000000000	U	2+	Enugu	6.450031351	7.499996703
NGA01000013-1031-2	285	4	■■■■■■■□□□□□■□□□□□□□□□□□□□□□□□□□□□□□□□□□□□□	NEW5	774040000000000	U	2+	Enugu	6.450031351	7.499996703
NGA01000013-010-1	293	4	■■■■■■■□□□□□■□□□□□□□□□□□□□□□□□□□□□□□□□□□□□□	NEW5	774040000000000	U	2+	Enugu	6.450031351	7.499996703
NGA01000027-325-2	502	6	■■■■■■■□□□□□□□□□□□□□□■■■■■■■■■■■■■■■□□□■■■■	new-n	774000077777071	Afri_2	3+	Ogun	7.160427265	3.350017455
NGA01000018-504-1	452	5	■■■■■■□□■■■■■■■■■■■■■■□□□■■■■■■■■□□□□■■■■■■■	new-m	771777743760771	T	2+	Kaduna	10.52001548	7.440000365
NGA010000037-567-1	924	11	■■■■■■□□■■■■■■■■■□□□□■■■■■■■■□□□□■■■■■■■	1064	771777607760771	LAM9	3+	Federal Capital Territory	9.083333149	7.533328002
NGA01000027-479-3	526	6	■■■■■■□□■■■■■■■■■□□□□■■■■■■■■□□□□■■■■■■■	1064	771777607760771	LAM9	3+	Ogun	7.160427265	3.350017455

NGA01000002-405-2	62	1	■■■■■■□□□■■■■■■■■■■■■■■■■■■■■■■■■■■■■■□■■■■	181	770777777777671	AFRI_1	2+	Adamawa	10.2703408	13.2700321
NGA01000011-1911-2	234	3	■■■■■■□□□■■■■■■■■■■■■■■■■■■■■■■■■■■■■■□■■■■	181	770777777777671	AFRI_1	2+	Ebonyi	6.5218	8.1298
NGA01000011-2010-2	274	3	■■■■■■□□□■■■■■■■■■■■■■■■■■■■■■■■■■■■■■□■■■■	181	770777777777671	AFRI_1	3+	Ebonyi	6.5218	8.1298
NGA01000012-2539-3	198	3	■■■■■■□□□■■■■■■■■■■■■■■■■■■■■■■■■■■■■■□■■■■	181	770777777777671	AFRI_1	3+	Edo	6.340477314	5.620008096
NGA010000030-265-2	722	9	■■■■■■□□□■■■■■■■■■■■■■■■■■■■■■■■■■■■■■□■■■■	181	770777777777671	AFRI_1	3+	Osun	7.480433572	4.560021117
NGA010000034-599-1	862	10	■■■■■■□□□■■■■■■■■■■■■■■■■■■■■■■■■■■■■■□■■■■	181	770777777777671	AFRI_1	3+	Taraba	8.900372741	11.36001949
NGA010000036-134-3	803	10	■■■■■■□□□■■■■■■■■■■■■■■■■■■■■■■■■■■■■■□■■■■	181	770777777777671	AFRI_1	2+	Zamfara	12.1704057	6.659996296
NGA010000036-361-2	818	10	■■■■■■□□□■■■■■■■■■■■■■■■■■■■■■■■■■■■■■□■■■■	181	770777777777671	AFRI_1	3+	Zamfara	12.1704057	6.659996296
NGA01000012-851-2	247	3	■■■■■■□□□■■■■■■■■■■■■□□□■■■■■■■■■■■■■■□■■■■	326	770777707777671	AFRI_1	2+	Edo	6.340477314	5.620008096
NGA010000026-995-2	673	8	■■■■■■□□□■■■■■■□■■■■■■■■■■■■■■■■■■■■■■□■■■■	2442	770773777777671	AFRI_1	3+	Niger	9.080413431	6.01001013
NGA01000021-463-1	463	5	■■■■■■□□□□■■■■■■■■■■■■■■■■■■■■■■■■■■■■□■■■■	1954	770377777777671	AFRI_1	2+	Kebbi	12.45041445	4.199939737
NGA01000006-724-1	172	2	■■■■■■□□□□□□□□□□■■■■■□□□□■■■■■■■■■■■■□□□■■■■	NEW8*	770003607777071	Afri_2	3+	Bayelsa	4.664030	6.036987
NGA01000010-1045-2	202	3	■■■■■■□□□□□□□□□□■■■■■□□□□■■■■■■■■■■■■□□□■■■■	NEW8*	770003607777071	Afri_2	3+	Delta	5.519958922	5.759999551
NGA01000012-2322-2	261	3	■■■■■■□□□□□□□□□□■■■■■□□□□■■■■■■■■■■■■□□□■■■■	NEW8*	770003607777071	Afri_2	3+	Edo	6.340477314	5.620008096
NGA01000017-166-2	418	5	■■■■■■□□□□□□□□□□■■■■■□□□□■■■■■■■■■■■■□□□■■■■	NEW8*	770003607777071	Afri_2	3+	Jigawa	11.7991891	9.350334607
NGA010000028-048-1	689	8	■■■■■■□□□□□□□□□□■■■■■□□□□■■■■■■■■■■■■□□□■■■■	NEW8*	770003607777071	Afri_2	3+	Ondo	7.530430521	5.759999551
NGA010000028-018-1	712	9	■■■■■■□□□□□□□□□□■■■■■□□□□■■■■■■■■■■■■□□□■■■■	NEW8*	770003607777071	Afri_2	2+	Ondo	7.530430521	5.759999551
NGA01000014-072-1	357	4	■■■■■■□□□□□□□□□□■■■■■□□□□■■■■■■■■■■■■□□□□□□■	NEW7'*	770003607777001	Afri_2	3+	Ekiti	7.630372741	5.219980834
NGA010000030-033-1	728	9	■■■■■■□□□□□□□□□□■■■■■□□□□■■■■■■■■■■■■□□□□□□■	NEW7'*	770003607777001	Afri_2	2+	Osun	7.480433572	4.560021117
NGA01000008-405-1	661	8	■■■■■■□□□□□□□□□□■■■■■□□□□■■□□■■■■■■■■□□□■■■■	320	770003606377071	Afri_2	3+	Borno	11.84996014	13.16001298
NGA01000010-1022-2	273	3	■■■■■■□□□□□□□□□□■■■■■□□□□■■□□■■■■■■■■□□□■■■■	320	770003606377071	Afri_2	3+	Delta	5.519958922	5.759999551
NGA01000011-2055-3	195	3	■■■■■■□□□□□□□□□□■■■■■□□□□■■□□■■■■■■■■□□□■■■■	320	770003606377071	Afri_2	3+	Ebonyi	6.5218	8.1298
NGA01000011-1673-2	204	3	■■■■■■□□□□□□□□□□■■■■■□□□□■■□□■■■■■■■■□□□■■■■	320	770003606377071	Afri_2	3+	Ebonyi	6.5218	8.1298
NGA01000012-2299-2	245	3	■■■■■■□□□□□□□□□□■■■■■□□□□■■□□■■■■■■■■□□□■■■■	320	770003606377071	Afri_2	3+	Edo	6.340477314	5.620008096
NGA01000014-195-3	311	4	■■■■■■□□□□□□□□□□■■■■■□□□□■■□□■■■■■■■■□□□■■■■	320	770003606377071	Afri_2	2+	Ekiti	7.630372741	5.219980834
NGA01000014-197-2	319	4	■■■■■■□□□□□□□□□□■■■■■□□□□■■□□■■■■■■■■□□□■■■■	320	770003606377071	Afri_2	3+	Ekiti	7.630372741	5.219980834
NGA01000014-203-3	327	4	■■■■■■□□□□□□□□□□■■■■■□□□□■■□□■■■■■■■■□□□■■■■	320	770003606377071	Afri_2	2+	Ekiti	7.630372741	5.219980834
NGA010000032-562-1	788	9	■■■■■■□□□□□□□□□□■■■■■□□□□■■□□■■■■■■■■□□□■■■■	320	770003606377071	Afri_2	2+	Rivers	4.810002257	7.010000772
NGA01000007-592-2	141	2	■■■■■□□□■■■■■■■■■■■■■□□■■■■■■■□□□□■■■□■■■■	844	761777743760731	CAM	2+	Benue	7.729979064	8.530011351
NGA01000007-493-2	142	2	■■■■■□□□■■■■■■■■■■■■■□□■■■■■■■□□□□■■■□■■■■	844	761777743760731	CAM	2+	Benue	7.729979064	8.530011351
NGA01000011-2200-1	259	3	■■■■■□□□■■■■■■■■■■■■■□□■■■■■■■□□□□■■■□■■■■	844	761777743760731	CAM	3+	Ebonyi	6.5218	8.1298
NGA01000010-1011-2	257	3	■■■□■■■■■■■■■■■■■■■■■■■■■■■■■■■□□□□■■■□■■■■	848	737777777760731	T2	3+	Delta	5.519958922	5.759999551
NGA01000015-1558-2	281	4	■■■□■■■■■■■■■■■■■■■■■■■■■■■■■■■□□□□■■■□■■■■	848	737777777760731	T2	3+	Gombe	10.29044293	11.16995357
NGA010000031-682-3	732	9	■■■□■■■■■■■■■■■■■■■■■■■■■■■■■■■□□□□■■■□■■■■	848	737777777760731	T2	3+	Plateau	9.929973978	8.890041055
NGA01000018-408-3	388	5	■■■□■■■■■■■■■■■■■■■■■■■■■■□■■■■■□□□□■■■□■■■■	new-l	737777777660731	T2	3+	Kaduna	10.52001548	7.440000365
NGA01000009-3529-3	185	2	■■■□■■■□□□□□□■■■■■■■□□□□■■■■■■■■■■■■□□□■■■■	NEW4	734077607777071	Afri_2	3+	Cross River	4.960406513	8.330023558
NGA01000011-1620-2	196	3	■■■□■■■□□□□□□■■■■■■■□□□□■■■■■■■■■■■■□□□■■■■	NEW4	734077607777071	Afri_2	3+	Ebonyi	6.5218	8.1298
NGA01000003-1247-1	2	1	■■■□□□□■■■■■■■■■■■■■■■■□□□□□□□□□□□□■■□□■■■■■	25	703777740003171	CAS1-Delhi	3+	Anambra	6.210433572	7.06999711
NGA01000002-212-1	8	1	■■■□□□□□□□□□□□□□■■■■■■■■■■■■■■■■■■■■■■□■■■■	new-k	700000377777671	AFRI_1	3+	Adamawa	10.2703408	13.2700321
NGA010000021-308-2	923	11	■■■□□□□□□□□□□□□□□□□□□■■■■■■■■■■■■■■■□□□■■■■	Orphan	700000077777071	Orphan	2+	Kebbi	12.45041445	4.199939737
NGA01000021-170-3	431	5	■■□■■■■■■■■■■■■■■■■■■■■■■■■■□□□□■□■■■■■■■■■	342	677777777413771	EAI5	3+	Kebbi	12.45041445	4.199939737
NGA010000035-201-2	817	10	■■□■■■■■■■■■■■■■■■■■■■■■■■■■□□□□■□■■■■■■■■■	342	677777777413771	EAI5	3+	Yobe	11.74899608	11.96600457
NGA010000030-071-1	911	11	■■□■■■■■■■■■■■■■■■■■■■■■■■■■□□□□□□■■■■□■■■■	526	6,77778E+14	Orphan	3+	Osun	7.480433572	4.560021117
NGA01000024-187-1	474	6	■■□■■■■■■■■■■■■■■■■□□■■■■■■□□□□□□■□■■■■■■■■■	895	677777477013771	EAI2_MANILLA	3+	Lagos	6.443261653	3.391531071
NGA01000022-910-3	440	5	■■□■■■■■■■■■■■■□□■■■■■■□□□□□□□□□□□□□□■□■■■	new-j	677774760000131	T	3+	Kogi	7.110404479	6.739939737
NGA010000034-584-1	847	10	■■□■■■■■□■■■■■■□■■■■■□■■■■■■■■□■■□■■■■■□□□□	Orphan	676773677667600	BOV	2+	Taraba	8.900372741	11.36001949
NGA01000014-286-1	358	4	■■□■■■■□□□□□□■■■■■□□□□■■■■■■■□□□■■■■■■■	2428	674017607760771	LAM3	3+	Ekiti	7.630372741	5.219980834
NGA010000026-029-3	672	8	■■□■■□■■□■■■■■■□■■■■■■■■■■■■■□■■■■■■■□□□□□□	1603	666773777677400	BOV_1	2+	Niger	9.080413431	6.01001013
NGA01000005-090-2	563	7	■□■■■■■■■■■■■■■■■■■■■■■■■■■■■■■■□□□□■■■■■■■	334	577777777760771	T1	3+	Bauchi	10.3103642	9.84000891
NGA010000028-010-2	626	8	■□■■■■■■■■■■■■■■■■■■■■■■■■■■■■■■□□□□■■■■■■■	334	577777777760771	T1	2+	Ondo	7.530430521	5.759999551
NGA010000029-238-1	604	7	■□■■■■■■■■■■■■■■■■■■■■□□□■■■■■■■□□□□■■■■■■■	new-f*	577777743760771	CAM	3+	Oyo	7.380026264	3.929982054
NGA010000035-1040-1	824	10	■□■■■■■■■■■■■■■■■■■■■■□□□■■■■■■■□□□□■■■■■■■	new-f*	577777743760771	CAM	2+	Yobe	11.74899608	11.96600457
NGA01000001-571-1	683	8	■□■■■■■□□□□□□■■■■■■■□□□□■■■■■■■■■■■■□□□■■■■	319	574077607777071	Afri_2	3+	Abia	5.100397968	7.34998002
NGA01000016-942-2	314	4	■□■■■■■□□□□□□■■■■■■■□□□□■■■■■■■■■■■■□□□■■■■	319	574077607777071	Afri_2	3+	Imo	5.492997053	7.026003588
NGA01000016-1176-2	315	4	■□■■■■■□□□□□□■■■■■■■□□□□■■■■■■■■■■■■□□□■■■■	319	574077607777071	Afri_2	3+	Imo	5.492997053	7.026003588
NGA01000021-514-1	439	5	■□■■■■■□□□□□□■■■■■■■□□□□■■■■■■■■■■■■□□□■■■■	319	574077607777071	Afri_2	2+	Kebbi	12.45041445	4.199939737
NGA01000022-394-1	449	5	■□■■■■■□□□□□□■■■■■■■□□□□■■■■■■■■■■■■□□□■■■■	319	574077607777071	Afri_2	2+	Kogi	7.110404479	6.739939737
NGA010000030-070-3	792	9	■□■■■■■□□□□□□■■■■■■■□□□□■■■■■■■■■■■■□□□■■■■	319	574077607777071	Afri_2	2+	Osun	7.480433572	4.560021117

NGA01000011-1958-2	250	3	■❑■■■■❑❑❑■■■■■■■■■■■■❑❑❑■■■■■■■■■■■■■■❑■■■■	NEW3	570777707777671	AFRI_1	2+	Ebonyi	6.5218	8.1298
NGA01000029-132-2	609	7	■❑■■■■❑❑❑■■■■■■■■■■■■❑❑❑■■■■■■■■■■■■■■❑■■■■	NEW3	570777707777671	AFRI_1	3+	Oyo	7.380026264	3.929982054
NGA01000010-1151-1	224	3	■❑■■❑■■■■■■■■■■■■■■■■■■■■■■■■■■■❑❑❑❑■■■■■■■	new-i	557777777760771	T	2+	Delta	5.519958922	5.759999551
NGA01000019-923-2	398	5	■❑■■❑■■■■■■■■■■■■■■■■■■■■■■■■■❑■❑❑❑❑■■■❑■■■	new-i	557777777720731	T	3+	Kano	11.99997683	8.5200378
NGA01000018-177-1	451	5	■❑■■❑■■■■■■■■■■■■■■■■■❑❑❑■■■■■■■❑❑❑❑■■■■■■■	NEW2	557777743760771	CAM	2+	Kaduna	10.52001548	7.440000365
NGA01000021-056-3	375	5	■❑■■❑■■■■■■■■■■■■■■■■■❑❑❑■■■■■■■❑❑❑❑■■■■■■■	NEW2	557777743760771	CAM	3+	Kebbi	12.45041445	4.199939737
NGA01000027-320-1	486	6	■❑■■❑■■■■■■■■■■■■■■■■■❑❑❑■■■■■■■❑❑❑❑■■■❑■■■	new-h	557777743760731	CAM	3+	Ogun	7.160427265	3.350017455
NGA01000027-216-3	534	6	■❑■■❑■■■■■■■■■■■■■■■■■❑❑❑■■■■■■❑❑❑❑❑■■■■■■■	NEW1	557777743740771	CAM	2+	Ogun	7.160427265	3.350017455
NGA01000027-237-1	550	6	■❑■■❑■■■■■■■■■■■■■■■■■❑❑❑■■■■■■❑❑❑❑❑■■■■■■■	NEW1	557777743740771	CAM	2+	Ogun	7.160427265	3.350017455
NGA010000037-335-2	859	10	■❑■■❑■■■■■■■■■■■■■■■■■❑❑❑■■■■■■❑❑❑❑❑■■■■■❑■	NEW1	557777743740771	CAM	2+	Federal Capital Territory	9.083333149	7.533328002
NGA010000036-335-1	865	10	■❑■■❑■■■■■■■■■■■■■■■■■❑❑❑■■■■■■❑❑❑❑❑■■■■■❑■	NEW1	557777743740771	CAM	3+	Zamfara	12.1704057	6.659996296
NGA01000019-3597-2	389	5	■❑■■❑■❑❑❑■■■■■■■■■■■■■■■■■■■■■■■■■■■■■❑■■■■	new-g	550777777777671	AFRI_1	3+	Kano	11.99997683	8.5200378
NGA01000021-483-2	400	5	■❑■■❑❑❑❑■■■■■■■■■■■■■■❑❑❑■■■■■■❑❑❑❑❑❑❑❑❑■■■	new-e	541777743740031	CAM	2+	Kebbi	12.45041445	4.199939737
NGA01000019-1007-2	461	5	■❑❑■■■■■■■■■■■■■■■■■■■■■■■■■■■❑■❑❑❑❑■■■■■■■	655	477777777720771	H3	3+	Kano	11.99997683	8.5200378
NGA01000006-566-2	163	2	■❑❑■■■■■■■■■■■■■■■■■■■❑❑❑■■■■■■■❑❑❑❑■■■■■■■	new-d	477777743760771	CAM	3+	Bayelsa	4.664030	6.036987
NGA01000021-351-3	424	5	■❑❑■■■■❑❑❑❑❑■■■■■■■■■■■■■■■■■■■■■■■■❑❑❑■■■■	new-c	474077777777071	Afri_2	2+	Kebbi	12.45041445	4.199939737
NGA01000012-3006-1	213	3	■❑❑■■■■❑❑❑❑❑■■■■■■■■❑❑❑❑■■■■■■❑■■■■❑❑❑❑■■■■	new-b	474077607736071	Afri_2	3+	Edo	6.340477314	5.620008096
NGA01000008-965-2	152	2	■❑❑❑❑❑❑❑❑❑❑❑❑❑❑❑■■■■■■❑❑❑■■■■■■■❑❑❑❑■■■■■■■	852	400003743760771	CAM	2+	Borno	11.84996014	13.16001298
NGA01000009-3505-3	161	2	■❑❑❑❑❑❑❑❑❑❑❑❑❑❑❑■■■■■■❑❑❑■■■■■■■❑❑❑❑■■■■■■■	852	400003743760771	CAM	3+	Cross River	4.960406513	8.330023558
NGA01000009-3843-2	162	2	■❑❑❑❑❑❑❑❑❑❑❑❑❑❑❑■■■■■■❑❑❑■■■■■■■❑❑❑❑■■■■■■■	852	400003743760771	CAM	2+	Cross River	4.960406513	8.330023558
NGA01000021-471-3	384	5	■❑❑❑❑❑❑❑❑❑❑❑❑❑❑❑■■■■■■❑❑❑■■■■■■■❑❑❑❑■■■■■■■	852	400003743760771	CAM	2+	Kebbi	12.45041445	4.199939737
NGA01000022-086-3	401	5	■❑❑❑❑❑❑❑❑❑❑❑❑❑❑❑■■■■■■❑❑❑■■■■■■■❑❑❑❑■■■■■■■	852	400003743760771	CAM	3+	Kogi	7.110404479	6.739939737
NGA01000024-752-2	467	6	■❑❑❑❑❑❑❑❑❑❑❑❑❑❑❑■■■■■■❑❑❑■■■■■■■❑❑❑❑■■■■■■■	852	400003743760771	CAM	3+	Lagos	6.443261653	3.391531071
NGA010000032-998-2	734	9	■❑❑❑❑❑❑❑❑❑❑❑❑❑❑❑■■■■■■❑❑❑■■■■■■■❑❑❑❑■■■■■■■	852	400003743760771	CAM	3+	Rivers	4.810002257	7.010000772
NGA010000033-025-3	821	10	■❑❑❑❑❑❑❑❑❑❑❑❑❑❑❑■■■■■■❑❑❑■■■■■■■❑❑❑❑■■■■■■■	852	400003743760771	CAM	3+	Sokoto	13.06001548	5.240031289
NGA01000010-309-3	201	3	❑■■■■■❑❑❑❑❑❑❑❑❑❑■■❑■❑❑❑❑■■■■❑❑❑❑❑❑❑❑❑❑❑■❑■■	new-a	370003207400051	Afri_2	3+	Delta	5.519958922	5.759999551
NGA01000015-638-1	321	4	❑❑❑■■■■■■■■■■■■■■■■■■■■■■■■■■■❑■❑❑❑■■■■❑■■■	307	077777777720731	H3	2+	Gombe	10.29044293	11.16995357
NGA01000015-1415-2	320	4	❑❑❑■■■■■■■■■■■■■■■■■■■■■■■■■■■❑■❑❑❑❑■■■❑■■■	307	077777777720731	H3	3+	Gombe	10.29044293	11.16995357
NGA01000026-1562-2	694	8	❑❑❑❑❑❑❑❑❑❑❑❑❑❑❑❑❑❑❑❑❑❑❑❑❑❑❑❑❑❑❑■❑❑❑❑■■■■■■■	47	000000000020771	H3	2+	Niger	9.080413431	6.01001013
NGA01000004-619-1	76	1	❑❑❑❑❑❑❑❑❑❑❑❑❑❑❑❑❑❑❑❑❑❑❑❑❑❑❑❑❑❑❑❑❑❑■■■■■■■■■	1	000000000003771	Beijing	2+	Akwa Ibom	5.007996056	7.849998524
