# Supplementary material for: Mycobacterium tuberculosis complex genotypes circulating in Nigeria based on spoligotyping obtained from Ziehl-Neelsen stained slides extracted DNA
Source: PLoS Negl Trop Dis. 2018 Feb 15;12(2):e0006242. doi: 10.1371/journal.pntd.0006242 (PMC5831734; doi:10.1371/journal.pntd.0006242)
Supplement: S2 Table — (PDF) [file pntd.0006242.s002.pdf]

Supplementary Table 2 : Country name, State name, Position, Capital, Lat (Y), Long (X), total sample n° (ACN), Spoligotype Lineage distribution (L1- L6 with L4 split into L462 (CAM) and L4OTHER (other L4), population, density, source of information of population data

Country	State	Position	Y	X	Capital	ACN	L1	L2	L3	L4	L462	L4OTHER	L5	L6	U	POPULATION	DENSITY*	Source population (accessed Dec. 8th 2017)
NGA	Abia	SE	5,524727	7,491903	Umuahia	19				13	4	9	4		2	3 727 300,00	500-600	https://www.citypopulation.de/Nigeria-Agglos.html?admid=5729
NGA	Adamawa	NE	9,203496	12,49539	Yola	10				8	2	6		2		4 248 400,00	70-100	https://www.citypopulation.de/Nigeria-Agglos.html?admid=5730
NGA	Akwa Ibom	SE	5,038963	7,90947	Uyo	7		1		3	1	2	3			5 482 200,00	500-600	https://www.citypopulation.de/Nigeria-Agglos.html?admid=5731
NGA	Anambra	SE	6,210528	7,072277	Awka	11			1	9	0	9	1			5 527 800,00	800-2000	https://www.citypopulation.de/Nigeria-Agglos.html?admid=5732
NGA	Bauchi	NE	10,314159	9,846282	Bauchi	3				3	1	2				6 537 300,00	70-100	https://www.citypopulation.de/Nigeria-Agglos.html?admid=5733
NGA	Bayelsa	SE	4,9142671	6,2752752	Yenagoa	12	1			7	6	1	4			2 278 000,00	150-200	https://www.citypopulation.de/Nigeria-Agglos.html?admid=5734
NGA	Benue	SE	7,7290834	8,4858854	Makurdi	17				9	5	4	8			5 741 800,00	100-150	https://www.citypopulation.de/Nigeria-Agglos.html?admid=5735
NGA	Borno	NE	11,8411223	12,9915464	Maiduguri	14				12	7	5	2			5 860 200,00	50-70	https://www.citypopulation.de/Nigeria-Agglos.html?admid=5736
NGA	Cross River	SE	4,974786	8,2703447	Calabar	11				6	5	1	5			3 866 000,00	100-150	https://www.citypopulation.de/Nigeria-Agglos.html?admid=5737
NGA	Delta	SE	6,2019032	6,6367999	Asaba	20				13	9	4	7			5 663 400,00	200-250	https://www.citypopulation.de/Nigeria-Agglos.html?admid=5738
NGA	Ebonyi	SE	6,3221162	8,0276554	Abakaliki	22				13	12	1	6	3		2 880 400,00	250-300	https://www.citypopulation.de/Nigeria-Agglos.html?admid=5739
NGA	Edo	SW	6,3477411	5,5724256	Benin City	23				16	11	5	5	1	1	4 235 600,00	150-200	https://www.citypopulation.de/Nigeria-Agglos.html?admid=5740
NGA	Ekiti	SW	7,6303004	5,1730985	Ado Ekiti	15				8	6	2	5		2	3 270 800,00	350-400	https://www.citypopulation.de/Nigeria-Agglos.html?admid=5741
NGA	Enugu	SE	6,4383753	7,4684256	Enugu	20				5	5	0	15			4 411 100,00	400-500	https://www.citypopulation.de/Nigeria-Agglos.html?admid=5742
NGA	Federal Capital Territory	Centre	9,0546462	7,254269	Abuja	14				13	12	1	1			3 564 100,00	70-100	https://www.citypopulation.de/Nigeria-Agglos.html?admid=5743
NGA	Gombe	NE	10,2885659	11,1435369	Gombe	15				15	9	6				3 257 000,00	100-150	https://www.citypopulation.de/Nigeria-Agglos.html?admid=5744
NGA	Imo	SE	5,5010524	6,9911663	Owerri	6				2	1	1	4			5 408 800,00	600-800	https://www.citypopulation.de/Nigeria-Agglos.html?admid=5745
NGA	Jigawa	NE	11,6982085	9,3055908	Dutse	21				19	15	4	2			5 828 200,00	150-200	https://www.citypopulation.de/Nigeria-Agglos.html?admid=5746
NGA	Kaduna	Centre	10,5072376	7,3592597	Kaduna	17				17	12	5				8 252 400,00	50-70	https://www.citypopulation.de/Nigeria-Agglos.html?admid=5747
NGA	Kano	NE	11,9981883	8,4008114	Kano	18				15	12	3	1	1	1	13 076 900,00	400-500	https://www.citypopulation.de/Nigeria-Agglos.html?admid=5748
NGA	Katsina	NW	12,9850601	7,5534071	Katsina	16				16	8	8				7 831 300,00	200-250	https://www.citypopulation.de/Nigeria-Agglos.html?admid=5749
NGA	Kebbi	NW	12,4514358	4,1801212	Birnin Kebbi	17	1			11	9	2	2	2	1	4 400 000,00	70-100	https://www.citypopulation.de/Nigeria-Agglos.html?admid=5750
NGA	Kogi	Centre	7,8036443	6,6988699	Lokoja	15				13	10	3	2			4 473 500,00	100-150	https://www.citypopulation.de/Nigeria-Agglos.html?admid=5751
NGA	Kwara	Centre	8,4785203	4,484916	Ilorin	16				15	11	4	1			3 192 900,00	50-70	https://www.citypopulation.de/Nigeria-Agglos.html?admid=5752
NGA	Lagos	SW	6,5488106	3,1173002	Lagos	17	1			15	11	4	1			12 550 600,00	>2000	https://www.citypopulation.de/Nigeria-Agglos.html?admid=5753
NGA	Nassarawa	Centre	8,5027488	8,5011433	Lafia	3				2	2	0	1			2 523 400,00	50-70	https://www.citypopulation.de/Nigeria-Agglos.html?admid=5754
NGA	Niger	Centre	9,6033597	6,4504233	Minna	22				20	7	13		2		5 556 200,00	40-50	https://www.citypopulation.de/Nigeria-Agglos.html?admid=5755
NGA	Ogun	SW	7,153389	3,2974403	Abeokuta	16				15	12	3	1			5 217 700,00	200-250	https://www.citypopulation.de/Nigeria-Agglos.html?admid=5756
NGA	Ondo	SW	7,254124	5,1198405	Akure	13				10	5	5	2		1	4 671 700,00	200-250	https://www.citypopulation.de/Nigeria-Agglos.html?admid=5757
NGA	Osun	SW	7,7865237	4,470467	Osogbo	16				12	11	1	2	1	1	4 705 600,00	350-400	https://www.citypopulation.de/Nigeria-Agglos.html?admid=5758
NGA	Oyo	SW	7,4211334	3,7640927	Ibadan	5				4	2	2		1		7 840 900,00	150-200	https://www.citypopulation.de/Nigeria-Agglos.html?admid=5759
NGA	Plateau	Centre	9,9155163	8,8032967	Jos	15				14	11	3	1			4 200 400,00	70-100	https://www.citypopulation.de/Nigeria-Agglos.html?admid=5760
NGA	Rivers	SE	4,8244929	6,8934581	Port Harcour	22				9	9	0	13			7 303 900,00	400-500	https://www.citypopulation.de/Nigeria-Agglos.html?admid=5761
NGA	Sokoto	NW	13,0324099	5,1578206	Sokoto	18				17	14	3			1	4 998 100,00	50-70	https://www.citypopulation.de/Nigeria-Agglos.html?admid=5762
NGA	Taraba	NE	8,9003332	11,3313667	Jalingo	18				15	13	2	1	2		3 066 800,00	40-50	https://www.citypopulation.de/Nigeria-Agglos.html?admid=5763
NGA	Yobe	NE	11,7471659	11,9501629	Damaturu	13	1			12	10	2				3 294 100,00	40-50	https://www.citypopulation.de/Nigeria-Agglos.html?admid=5764
NGA	Zamfara	NW	12,1809321	6,6344539	Gusau	12				8	6	2	1	2	1	4 515 400,00	70-100	https://www.citypopulation.de/Nigeria-Agglos.html?admid=5765

*population density was recorded using : http://www.iplussolutions.org/isolutions-leads-consortium-streamline-patient-access-essential-treatments-nigeria-0, assessed on November 2017, 20th
