# Supplementary material for: Mycobacterium tuberculosis complex genotypes circulating in Nigeria based on spoligotyping obtained from Ziehl-Neelsen stained slides extracted DNA
Source: PLoS Negl Trop Dis. 2018 Feb 15;12(2):e0006242. doi: 10.1371/journal.pntd.0006242 (PMC5831734; doi:10.1371/journal.pntd.0006242)
Supplement: S3 Table — (PDF) [file pntd.0006242.s003.pdf]

Supplementary Table 3: spoligotyping clusters, not found in SITVITWEB, but found between this study and Sharma *et al*. 2016, with total number of isolates concerned

Arbitrary pattern database number	Binary	n°	Cluster designation	Lineage designation
MAFUSA163	■■■■■■■□□□□□■■■■■■■■□□□□■■■■■■■■■■■■□□□■□□■	1	NEW9	L5/*Mycobacterium africanum* WA1
NGA064	■■■■■■■□□□□□■■■■■■■■□□□□■■■■■■■■■■■■□□□■□□■	2	NEW9	L5/*Mycobacterium africanum* WA1
MAFUSA285	■■■■■■■□□□□□■■■■■■■■□□□□■■■■■■■□□□□□□□□■■■■	1	NEW6	L5/*Mycobacterium africanum* WA1
NGA065	■■■■■■■□□□□□■■■■■■■■□□□□■■■■■■■□□□□□□□□■■■■	2	NEW6	L5/*Mycobacterium africanum* WA1
MAFUSA40	■■■■■■■□□□□□■■■■■■■■□□□□□□□□□□□□□□□□□□□■■■■	2	new-p	L5/*Mycobacterium africanum* WA1
NGA029	■■■■■■■□□□□□■■■■■■■■□□□□□□□□□□□□□□□□□□□■■■■	1	new-p	L5/*Mycobacterium africanum* WA1
MAFUSA281	■■■■■■■□□□□□■■■■■■■■□□□□□□□□□□□□□□□□□□□□■■■	1	NEW11*	L5/*Mycobacterium africanum* WA1
NGA089	■■■■■■■□□□□□■■■■■■■■□□□□□□□□□□□□□□□□□□□□■■■	5	NEW11*	L5/*Mycobacterium africanum* WA1
MAFUSA272	■■■■■■■□□□□□□□□□□□□□■■■■■■■■■■■■■■■□□□■■■■	1	new-n	L5/*Mycobacterium africanum* WA1
NGA032	■■■■■■■□□□□□□□□□□□□□■■■■■■■■■■■■■■■□□□■■■■	1	new-n	L5/*Mycobacterium africanum* WA1
MAFUSA5	■■■■■■□□□□□□□□□□■■■■□□□□■■■■■■■■■■■■□□□■■■■	8	NEW8*	L5/*Mycobacterium africanum* WA1
NGA092	■■■■■■□□□□□□□□□□■■■■□□□□■■■■■■■■■■■■□□□■■■■	6	NEW8*	L5/*Mycobacterium africanum* WA1
MAFUSA71	■■■■■■□□□□□□□□□□■■■■□□□□■■■■■■■■■■■■□□□□□□■	1	NEW7'*	L5/*Mycobacterium africanum* WA1
NGA067	■■■■■■□□□□□□□□□□■■■■□□□□■■■■■■■■■■■■□□□□□□■	2	NEW7'*	L5/*Mycobacterium africanum* WA1

NGA, Nigeria

USA, United States of America
